# IVF in endometriosis: emerging evidence of exacerbation of pelvic pain and potential predictors

**DOI:** 10.1093/hropen/hoag027

**Published:** 2026-03-27

**Authors:** Jonas Vibert, Eliane Maalouf, Jean-Marie Wenger, Antonio Mercorio, Giuseppe Benagiano, Nicola Pluchino

**Affiliations:** Department of Obstetrics and Gynecology, Lausanne University Hospital, Lausanne, Switzerland; Information Management Institute, University of Neuchâtel, Neuchâtel, Switzerland; Faculty of Medicine, University of Geneva, Geneva, Switzerland; Assisted Reproductive Technology Unit, Department of Gynecology, Hôpital Foch, Suresnes, France; Geneva Foundation for Medical Education and Research, Geneva, Switzerland; Faculty of Medicine and Dentistry, Sapienza University of Rome, Rome, Italy; Department of Obstetrics and Gynecology, Lausanne University Hospital, Lausanne, Switzerland

**Keywords:** endometriosis, IVF, reproductive techniques, assisted, pelvic pain, dysmenorrhoea, dyspareunia, pain measurement, patient-reported outcome measures

## Abstract

**STUDY QUESTION:**

Does IVF worsen pelvic pain in women with endometriosis?

**SUMMARY ANSWER:**

Nearly half of women with endometriosis reported perceived worsening of pelvic pain after IVF.

**WHAT IS KNOWN ALREADY:**

Prior studies generally suggested no IVF-related pain worsening, but few assessed delayed flares or longer-term trajectories.

**STUDY DESIGN, SIZE, DURATION:**

International cross-sectional study based on an online survey conducted between September 2024 and April 2025 including 546 respondents.

**PARTICIPANTS/MATERIALS, SETTING, METHODS:**

Women aged ≥18 years with surgically or imaging-confirmed endometriosis and at least one completed IVF cycle. A 25-item questionnaire captured demographics, reproductive history, comorbidities, and patient-reported pain trajectories before, during, and after IVF. The primary outcome was perceived worsening of pelvic pain after IVF (patient-reported outcome measure; PROM). Secondary outcomes were worsening of dysmenorrhoea and dyspareunia. Group comparisons and exploratory multivariable logistic regressions were performed. Predictor analyses were exploratory.

**MAIN RESULTS AND THE ROLE OF CHANCE:**

Among 546 respondents, 48.9% reported worsening pelvic pain after IVF, 49.1% reported worsening dysmenorrhoea, and 35.5% worsening dyspareunia. Current pain scores were significantly higher in women reporting worsening versus no worsening (all *P* < 0.001). In multivariable analyses, immediate post-cycle pain flare emerged as the strongest and most consistent predictor across all pain outcomes, independently associated with worsening of pelvic pain (adjusted odds ratio [aOR] 5.91, 95% CI 3.88–9.14), dysmenorrhoea (aOR 4.03, 95% CI 2.08–8.05), and dyspareunia (aOR 3.17, 95% CI 2.07–4.90) (all *P* < 0.001). For the primary outcome, reporting oocyte retrieval as the most painful IVF step (aOR 0.53, 95% CI 0.31–0.88; *P* = 0.016) and achieving a live birth after IVF (aOR 0.63, 95% CI 0.42–0.92; *P* = 0.020) were independently associated with lower odds of pelvic pain worsening. In secondary outcome models, live birth was associated with lower odds of dysmenorrhoea worsening, while bladder pain syndrome/interstitial cystitis independently predicted worsening of dyspareunia. A formal response rate could not be calculated due to open online dissemination without a known denominator.

**LIMITATIONS, REASONS FOR CAUTION:**

Self-reported, retrospective data are prone to recall and selection bias, and the cross-sectional design precludes causal inference. Recruitment via associations and social media without a denominator limits generalizability, absence of baseline pain scores impedes assessment of change, and incomplete capture of peri-IVF hormonal regimens may confound results.

**WIDER IMPLICATIONS OF THE FINDINGS:**

IVF may not be pain-neutral in endometriosis. Monitoring pain trajectories at key IVF milestones may help identify women at risk of long-term exacerbation. Prospective studies should test whether early monitoring combined with tailored interventions—optimized analgesia, psychological support, or adapted stimulation protocols—can mitigate chronic pain trajectories throughout the IVF journey and ultimately improve quality of life.

**STUDY FUNDING/COMPETING INTEREST(S):**

No specific funding; authors declare no conflicts of interest.

**TRIAL REGISTRATION NUMBER:**

N/A.

WHAT DOES THIS MEAN FOR PATIENTS? Endometriosis is a condition that can cause chronic pelvic pain and infertility. Many women with endometriosis undergo *in vitro* fertilization (IVF), but it is still unclear whether IVF can affect pain symptoms.

In this international online survey of 546 women with endometriosis who had undergone IVF, participants answered questions about their past IVF treatments and how their pain changed afterwards. About half reported that their pelvic pain worsened after IVF. Women who experienced a short-term flare of pain immediately after an IVF cycle were more likely to report longer-term worsening of pain. By contrast, when pain was mainly limited to the egg collection procedure, or when IVF resulted in a live birth, worsening of pelvic pain was less common.

These findings suggest that IVF may not be pain-neutral for all women with endometriosis. However, because this study relied on women recalling and reporting their past experiences, it cannot prove that IVF itself caused the pain to worsen. Monitoring pain symptoms during and after IVF may help clinicians identify women who might benefit from additional support. Future studies that follow women over time will help clarify these relationships and identify ways to reduce pain during fertility treatment.

## Introduction

Endometriosis is a chronic and often debilitating condition that affects ∼50% of women with subfertility, many of whom require IVF to achieve pregnancy (Garcia-Velasco and Somigliana, 2009; Kuznetsov *et al.*, 2017). Endometriosis, in addition to its association with subfertility and infertility, is characterized by debilitating pelvic pain, including dysmenorrhoea, dyspareunia, and chronic non-cyclic pain, which substantially impairs quality of life and presents significant challenges for effective clinical management (Fourquet *et al.*, 2011).

IVF remains a fundamental approach in managing infertility associated with endometriosis. Although an early study had already explored its emotional, physical, and relational impacts (Boivin and Takefman, 1996), the specific influence of IVF on endometriosis-related pain symptoms remains insufficiently characterized. Some studies have reported negative effects of IVF procedures on patients’ quality of life (Wu *et al.*, 2021; Zarbo *et al.*, 2023; Mori *et al.*, 2024), whereas others found no significant change in pain severity during or after treatment (Benaglia *et al.*, 2011; Santulli *et al.*, 2016; Somigliana *et al.*, 2019, 2025), with Cathelain *et al.* (2023) reporting higher baseline pain, without further increase during IVF.

Pain management in this context requires balancing effective symptom control with preservation of reproductive potential. Moreover, healthcare support remains a critical factor, with many women reporting dissatisfaction with counselling, pain management, and psychological support (Van Der Houwen, 2022).

Given the limited and heterogeneous evidence, the effect of IVF on endometriosis-related pain remains poorly defined. To address these gaps, we conducted a large international cross-sectional survey of women with endometriosis who had completed at least one IVF cycle. The primary objective was to quantify the perceived evolution of pelvic pain following IVF. Secondary objectives were to evaluate dysmenorrhoea and dyspareunia and to explore clinical, procedural, and psychosocial factors associated with symptom trajectories. Focusing on both measurement of perceived change through patient-reported outcome measures (PROMs) and exploratory prediction, we aimed to better characterize the endometriosis patient perspective throughout their IVF journey.

## Materials and methods

### Design and participants

We conducted a large, international, cross-sectional survey of women aged ≥18 years with surgically or imaging-confirmed endometriosis who had undergone at least one IVF cycle. A 25-item structured, web-based questionnaire captured sociodemographics, endometriosis history (age at diagnosis, diagnostic delay, prior surgery, revised American Society for Reproductive Medicine (rASRM) stage), fertility background (time to conception, prior deliveries), and details of all IVF cycles (country/setting, out-of-pocket costs, number of oocyte retrievals and embryo transfers, pregnancy and live-birth outcomes, complications). Items also assessed partner-related emotional/relational impact and satisfaction with medical care. To reduce recall bias, pain questions used event-based prompts linked to IVF milestones (ovarian stimulation, oocyte retrieval, embryo transfer, first menstruation after IVF), consistent with the peak-end rule (Schneider *et al.*, 2011). The full instrument is provided in Supplementary File S1. The reliability of self-reported endometriosis data in online surveys has been previously demonstrated (Gouesbet *et al.*, 2025).

Pain was assessed in two ways. (i) Perceived evolution since IVF using anchor-based PROM: participants rated overall pelvic pain, dyspareunia, and dysmenorrhoea on a 7-point Likert scale (‘much better’ to ‘much worse’). Assessing pain using broad ordinal categories instead of precise numerical scales reduces the influence of over- or underestimation and reflects the limited precision of retrospective self-reporting. For analysis, responses were dichotomized into worsening versus no worsening defining the primary outcome as binary. Secondary outcomes were perceived worsening of dyspareunia and dysmenorrhoea as binary. (ii) Current pain at survey completion: pelvic pain and dyspareunia were measured on a 0–10 visual analogue scale (VAS); dysmenorrhoea on a 0–10 VAS. Pre-IVF VAS were not collected.

This anchor-based PROM approach aligns with FDA PRO Guidance (2009) and contemporary PROM/MCID methodology, in which the minimal clinically important difference (MCID) is defined as the smallest change in an outcome that patients perceive as meaningful and that would justify a change in clinical management (McKenna, 2011; Kluzek *et al.*, 2022), rather than reliance on absolute changes in VAS scores alone (Huskisson, 1974).

### Recruitment and procedures

Recruitment occurred via patient advocacy organizations in Australia, France, Germany, Portugal, Spain, Sweden, Switzerland, the Netherlands, and the USA. To maximize accessibility, the questionnaire was available in French, German, English, Spanish, Portuguese, Italian, and Turkish. Data were collected from September 2024 to April 2025. Partner organizations were contacted by email and invited to distribute the survey link to their members.

### Statements of ethics

The study protocol was submitted to the Ethics Committee of the Canton of Vaud (CER-VD; Req-2024-01427), which determined that the project fell outside the scope of the Swiss Human Research Act and that formal approval was not required. Participation was voluntary, and electronic informed consent was obtained from all participants.

### Sample size calculation

We anticipated up to 25 candidate predictors and applied the events-per-variable (EPV) rule of ∼10 to limit overfitting (Ogundimu *et al.*, 2016). With an expected worsening pelvic pain prevalence of ∼50%, a sample of 500 participants (≈250 events) was targeted to ensure both precise prevalence estimates (95% CI ±4–5%) and adequate stability for exploratory multivariable models. The final sample (N = 546; ≈273 events, EPV ≈ 13.7) exceeded these thresholds. For illustration, classical power calculations indicate that 392 participants would suffice to detect an OR of 1.8 with 80% power (α = 0.05), further supporting adequacy of the achieved sample size.

### Statistical analysis

Analyses were performed in R v4.5.1 (R Foundation for Statistical Computing, Vienna, Austria). Demographics, clinical characteristics, and questionnaire responses were summarized descriptively: categorical variables as proportions (%) and continuous variables as means (SD) or medians (IQR), depending on distribution. Missing responses coded as ‘I don’t know/I don’t remember’ were retained as informative categories. No imputation was applied to outcome variables and regression modelling was performed with complete cases.

Group comparisons used χ^2^ or Fisher’s exact tests for categorical data and Wilcoxon rank-sum tests for continuous VAS scores. Univariable logistic regressions were conducted for candidate predictors of pain worsening, and clinically or statistically relevant variables were used in multivariable models for pelvic pain, dysmenorrhoea, and dyspareunia. Full results of the univariable analyses are presented in Supplementary Table S1. Results are reported as odds ratios (ORs) with 95% CIs. Model calibration was assessed with the Hosmer–Lemeshow test and multicollinearity with variance inflation factors. Age at first IVF cycle and time since first IVF cycle were included simultaneously to disentangle baseline age effects from the duration of the IVF trajectory; multicollinearity was assessed and found to be acceptable. All tests were two-sided, with *P* < 0.05 considered statistically significant.

## Results

### Sample characteristics

A total of 546 women with endometriosis who had completed ≥1 IVF cycle responded. Data on the primary outcome (pelvic pain evolution after IVF) were available for 530 participants. The mean age at the first IVF cycle was 33.8 years (SD 4.6). The mean time since the first IVF cycle was 3.9 years (SD 4.3). Most respondents were from Europe (78.0%) and held tertiary education (78.0%). Overall, 70.9% had undergone at least one endometriosis surgery; among those reporting revised ASRM (rASRM) stage, 69% were stage IV. The median number of IVF cycles was 2 (IQR 1–3). Across the cohort, 55.7% achieved at least one pregnancy and 35.9% at least one live birth. Comorbidities were frequent; Table 1 shows a condensed overview, with the full sample characteristics available in Supplementary Table S2.

**Table 1.** Baseline characteristics of participants stratified by self-reported pelvic pain evolution after IVF (N = 546).

Characteristic	Total (N = 546)	No worsening (N = 263)	Worsening (N = 267)	*P*-value
**Patient characteristics**				
Age at survey				0.516
≤30 years	33 (6.0%)	20 (7.6%)	13 (4.9%)	
31–35 years	145 (26.6%)	72 (27.4%)	69 (25.8%)	
36–40 years	226 (41.4%)	105 (39.9%)	118 (44.2%)	
>40 years	142 (26.0%)	66 (25.1%)	67 (25.1%)	
Age at first cycle (years)	33.8 (4.6)	33.3 (4.3)	34.1 (4.9)	0.100
Time since first IVF (years)	3.9 (4.3)	4.1 (4.6)	3.7 (3.9)	0.649
Geographic region				**0.018**
Europe	426 (78.0%)	218 (82.9%)	197 (73.8%)	
North America	82 (15.0%)	34 (12.9%)	45 (16.9%)	
Other	38 (7.0%)	11 (4.2%)	25 (9.4%)	
Education level				0.469
Primary school	17 (3.1%)	9 (3.4%)	8 (3.0%)	
Secondary education	101 (18.5%)	45 (17.1%)	52 (19.5%)	
Tertiary education	426 (78.0%)	207 (78.7%)	207 (77.5%)	
Unknown	2 (0.4%)	2 (0.8%)	0 (0.0%)	
Hormonal treatment	206 (37.7%)	98 (37.3%)	102 (38.2%)	0.823
**Comorbidities**				
Depression	150 (27.5%)	65 (24.7%)	80 (30.0%)	0.175
Anxiety disorder	158 (28.9%)	65 (24.7%)	88 (33.0%)	**0.036**
Chronic back pain	100 (18.3%)	46 (17.5%)	52 (19.5%)	0.556
Irritable bowel syndrome	124 (22.7%)	48 (18.3%)	72 (27.0%)	**0.017**
Bladder pain syndrome	32 (5.9%)	7 (2.7%)	23 (8.6%)	**0.003**
**Fertility characteristics**				
Birth before IVF	27 (4.9%)	8 (3.0%)	18 (6.7%)	**0.049**
Number of IVF cycles	2.0 (1.0, 3.0)	2.0 (1.0, 3.0)	2.0 (1.0, 3.0)	0.561
**Endometriosis characteristics**				
ASRM endometriosis stage				0.473
No surgery	159 (29.1%)	74 (28.1%)	78 (29.2%)	
ASRM I	10 (1.8%)	5 (1.9%)	5 (1.9%)	
ASRM II	27 (4.9%)	14 (5.3%)	12 (4.5%)	
ASRM III	56 (10.3%)	26 (9.9%)	29 (10.9%)	
ASRM IV	207 (37.9%)	94 (35.7%)	109 (40.8%)	
Unknown surgery	87 (15.9%)	50 (19.0%)	34 (12.7%)	
Deep infiltrating endometriosis	282 (51.6%)	127 (48.3%)	148 (55.4%)	**0.015**

Of the 546 participants, 16 did not respond to the primary outcome question (self-reported pelvic pain evolution after IVF) and could not be classified into either comparison group. These participants are included in the Total column but excluded from group comparisons and all regression analyses.

Data are presented as n (%) for categorical variables, mean (SD) for continuous variables, or median (Q1, Q3) for the number of IVF cycles.

ASRM, American Society for Reproductive Medicine; Q1, first quartile; Q3, third quartile.

Statistical tests: Pearson chi-squared test for categorical variables; Wilcoxon rank-sum test for continuous variables. Bold *P*-values indicate statistical significance (*P* < 0.05).

### Pain trajectories

**Figure 1.**
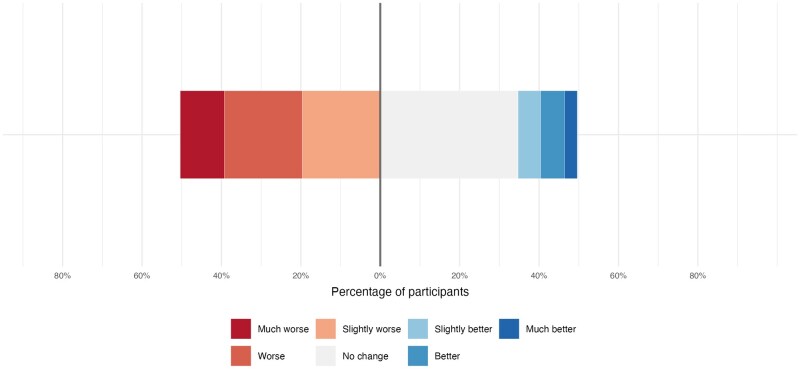
**Distribution of pelvic pain evolution after IVF among women with endometriosis**. Diverging bar chart showing the distribution of self-reported changes in pelvic pain following IVF treatment (n = 530). Pain evolution was assessed using a 7-point Likert scale ranging from ‘much worse’ to ‘much better’. Responses indicating worsening are displayed on the left side of the chart, while responses indicating improvement are displayed on the right side. ‘No change’ responses are shown on the right side for graphical balance.

**Figure 2.**
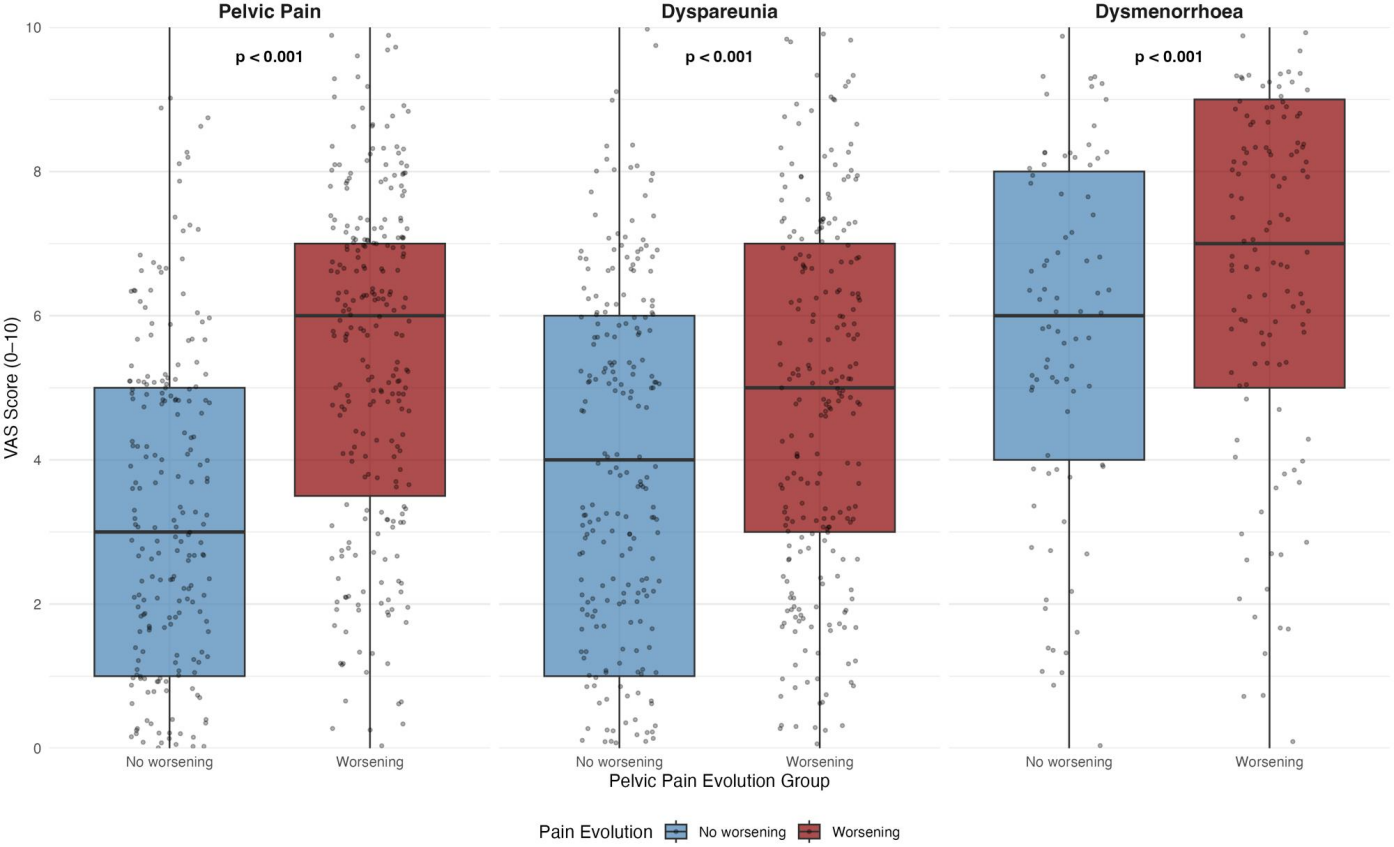
**Current pain intensity at survey completion according to self-reported pain evolution after IVF**. Boxplots showing current pain intensity measured using a visual analogue scale (VAS, 0–10) at the time of survey completion, stratified by self-reported evolution of pain after IVF. Three pain domains are presented: pelvic pain, dyspareunia, and dysmenorrhoea. Participants were grouped according to whether they reported worsening or no worsening of symptoms since IVF treatment. Boxes represent the interquartile range (IQR) with the median shown as a horizontal line. Whiskers represent the minimum and maximum values. Individual points correspond to participant-level observations. Group comparisons were performed using two-sided Wilcoxon rank-sum tests (Mann–Whitney U), computed separately for each pain domain. All comparisons were statistically significant (*P* <0.001). VAS, visual analogue scale.

Overall, 48.9% experienced worsening of pelvic pain after IVF, 35.5% of dyspareunia, and 49.1% of dysmenorrhoea. The distribution of pain evolution is displayed in Fig. 1, with additional detailed distributions for key predictors (immediate post-cycle flare, oocyte retrieval as the most painful step, and live birth following IVF) provided in Supplementary Fig. S1. At the time of the survey, 37.7% of participants were receiving hormonal treatment. Among these patients, 49.5% reported worsening pelvic pain after IVF, compared with 48.5% among those not receiving hormonal treatment. Current pain scores were higher in the ‘worsening’ versus ‘no-worsening’ group: median pelvic pain VAS 6.0 (IQR 3.0–7.0) versus 3.0 (IQR 1.0–5.0), *P* < 0.001 (Fig. 2).

### Potential predictors of pelvic pain worsening

**Figure 3.**
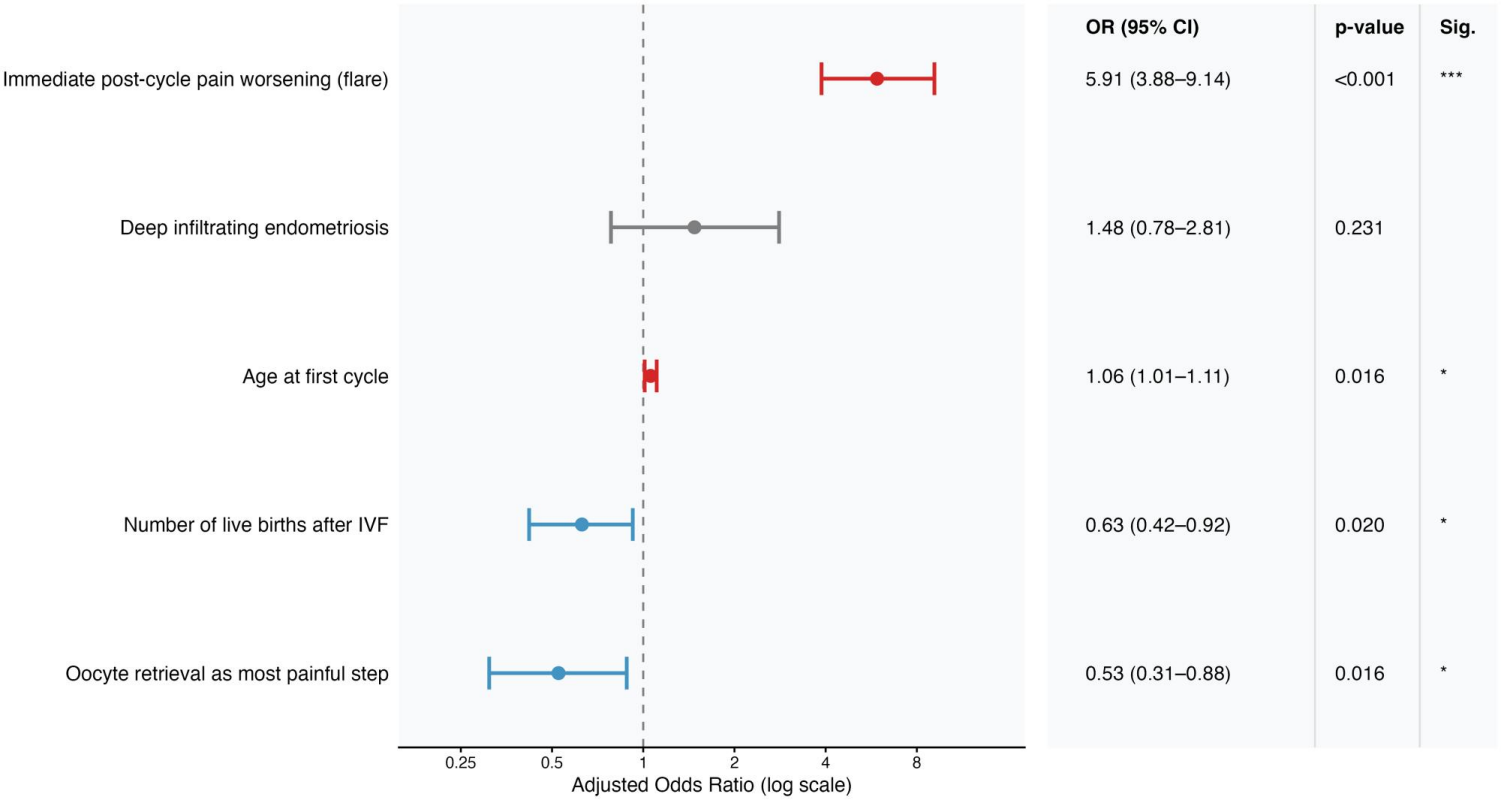
**Predictors associated with pelvic pain worsening after IVF**. Forest plot showing adjusted odds ratios (aOR) and 95% CIs from multivariable logistic regression analyses evaluating factors associated with worsening pelvic pain after IVF among women with endometriosis (n = 530). The vertical dashed line represents the null value (OR = 1). Odds ratios are displayed on a logarithmic scale. Red markers indicate significant risk factors (OR > 1), blue markers indicate significant protective factors (OR < 1), and grey markers indicate non-significant associations. Statistical significance is indicated as follows: *P < 0.05, ** *P* < 0.01, *** *P* < 0.001. OR, odds ratio.

In multivariable analysis (Table 2; Fig. 3), four factors showed independent associations with pelvic pain worsening: immediate post-cycle flare (aOR 5.91, 95% CI 3.88–9.14; *P* < 0.001); oocyte retrieval as the most painful step (aOR 0.53, 95% CI 0.31–0.88; *P* = 0.016); live birth after IVF (aOR 0.63, 95% CI 0.42–0.92; *P* = 0.020); and age at the first IVF cycle (aOR 1.06 per year, 95% CI 1.01–1.11; *P* = 0.016). rASRM stage, prior surgery, and the number of IVF cycles were not associated. Hormonal therapy status at the time of survey completion was not associated with pelvic pain worsening (aOR 1.03, 95% CI 0.67–1.58; *P* = 0.894).

**Table 2.** Multivariable logistic regression for predictors of pelvic pain worsening after IVF.

Predictor	OR	95% CI Lower	95% CI Upper	*P*-value	Sig.
**Patient characteristics**					
Education level (ref: Primary school)					
Secondary School	0.87	0.26	3.03	0.816	
Tertiary Education	0.85	0.27	2.79	0.780	
Age at first IVF cycle (per year)	1.06	1.01	1.11	**0.016**	*
Time since first IVF cycle (per year)	1.00	0.95	1.05	0.899	
Hormonal treatment	1.03	0.67	1.58	0.894	
**Comorbidities**					
Anxiety disorder	1.29	0.79	2.13	0.312	
Depression	1.14	0.69	1.89	0.601	
Bladder pain syndrome	1.91	0.71	5.72	0.219	
Irritable bowel syndrome	1.37	0.84	2.26	0.212	
**Fertility characteristics**					
Birth before IVF	1.46	0.57	3.99	0.445	
Number of live births	0.63	0.42	0.92	**0.020**	*
Number of miscarriages	0.81	0.57	1.17	0.265	
Number of IVF cycles	0.95	0.82	1.10	0.520	
Complications during IVF	1.11	0.50	2.48	0.792	
**Endometriosis characteristics**					
ASRM stage (ref: Stage I)					
No surgery	1.26	0.28	5.59	0.754	
Stage II	1.16	0.21	6.22	0.861	
Stage III	1.20	0.23	6.34	0.826	
Stage IV	1.18	0.24	5.74	0.836	
Unknown	0.95	0.20	4.47	0.944	
Deep infiltrating endometriosis	1.48	0.78	2.81	0.231	
**Pain characteristics**					
Most painful IVF step (ref: Ovarian stimulation)					
Trigger injection	0.97	0.38	2.50	0.948	
Oocyte retrieval	0.53	0.31	0.88	**0.016**	*
Embryo transfer	1.12	0.45	2.83	0.807	
Early pregnancy	1.18	0.36	3.85	0.779	
First menstruation after retrieval	1.29	0.66	2.55	0.467	
Don’t know	0.84	0.36	1.90	0.677	
Unbearable pain during IVF	0.74	0.27	2.02	0.552	
Immediate post-cycle pain worsening (flare)	5.91	3.88	9.14	**<0.001**	***

Of the 546 participants included in the descriptive analyses, 16 did not answer the primary outcome question and were excluded from regression analyses. Analyses of the primary outcome were therefore based on 530 participants; sample size may vary across predictors because of item-level missingness.

Results from multivariable logistic regression. Odds ratios (OR) and 95% CI are shown.

Reference categories: primary school education (Education level), ASRM stage I (endometriosis stage), and ovarian stimulation (most painful IVF step). Age at first IVF cycle was mean-centred prior to inclusion. For binary variables, the reference category is absence of the characteristic (No); for continuous variables, the OR reflects a one-unit increase.

ASRM, American Society for Reproductive Medicine; OR, odds ratio.

Bold *P*-values and significance markers indicate statistical significance (*P* < 0.05).

*P < 0.05;

**P < 0.01;

***P < 0.001.

### Potential predictors of dysmenorrhoea and dyspareunia

In adjusted models (Supplementary Tables S3 and S4), immediate post-cycle flare remained the strongest predictor for both outcomes (dysmenorrhoea: aOR 4.03, 95% CI 2.08–8.05; *P* < 0.001; dyspareunia: aOR 3.17, 95% CI 2.07–4.90; *P* < 0.001). Bladder pain syndrome/interstitial cystitis was independently associated with worsening dyspareunia (aOR 2.79, 95% CI 1.20–6.89; *P* = 0.020), but not with dysmenorrhoea. Live birth was associated with lower odds of dysmenorrhoea (aOR 0.47, 95% CI 0.23–0.89; *P* = 0.027).

### Patient experience

Among women with worsening pain, 50.2% reported dissatisfaction with medical services, 83.0% described a negative impact on mood, and 20.8% reported a high negative impact on partner relationships. Use of analgesics and complementary therapies was also higher (Supplementary Table S2).

## Discussion

Earlier studies found no increase in endometriosis-related pain after IVF (Benaglia *et al.*, 2011; Santulli *et al.*, 2016; Somigliana *et al.*, 2019, 2025; Cathelain *et al.*, 2023). In contrast, nearly half of our participants reported worsening pelvic pain when pain evolution was assessed through patient-perceived change using categorical variables, suggesting that a subset of women may be particularly susceptible to post-IVF pain exacerbation.

Immediate post-cycle flares emerged as the strongest potential predictor of long-term worsening, whereas reporting oocyte retrieval as the most painful step and achieving a live birth appeared protective. This pattern could suggest that pain confined to oocyte retrieval—the procedure most consistently associated with discomfort (Frederiksen *et al.*, 2017)—or attenuated by reproductive success reflects a more physiological trajectory, while persistent flares extending beyond the IVF period may represent a non-physiological response. Although speculative, this raises the hypothesis that women deviating from a ‘physiological pain pattern’ during IVF could be those most at risk of developing persistent pain. Event-anchored PROMs at predefined IVF milestones could identify at-risk patients earlier. Such monitoring would capture not only post-cycle flares but also retrieval-limited pain, transient peri-cycle discomfort, and persistent escalation. Identifying these patterns may enable stratified supportive strategies—including closer follow-up, optimized analgesia, and counselling—and improve understanding of distinct pain trajectories during IVF. These reflections remain exploratory and require prospective validation.

Older age at the first IVF cycle was also independently associated with pelvic pain worsening, although with a modest effect size, whereas anatomical disease severity was not independently associated with worsening, supporting the idea that factors beyond lesion burden—such as central sensitization and psychological distress—play an important role (Eriksen *et al.*, 2008; Laganà *et al.*, 2017; Song *et al.*, 2023). While mechanistic inferences cannot be drawn from this design, reproductive outcomes appeared influential. Live birth after IVF consistently reduced the risk of worsening, whereas prior spontaneous pregnancies did not. Miscarriage after IVF was also not associated with worsening, suggesting that the very achievement of conception—even if not sustained—may reassure women of their reproductive potential. The protective effect may therefore be more closely linked to the clinical and emotional impact of IVF success than to reproductive history alone. Current hormonal therapy at survey completion was not independently associated with pain worsening, suggesting that the observed trajectories were not driven by hormonal suppression status at the time of reporting.

Finally, patient experience deserves emphasis. Nearly half of respondents reported that medical services did not adequately address their pain and emotional needs, echoing previous findings (Van Der Houwen, 2022). The high prevalence of anxiety and depression in our cohort, consistent with earlier studies (Szypłowska *et al.*, 2023; Zippl *et al.*, 2024), together with the frequent use of complementary therapies, highlights both the physical and emotional burden carried by this population and the potential unmet supportive needs.

### Limitations

This study should be interpreted as exploratory. Its retrospective, self-reported design exposes results to recall and reporting bias, while recruitment via patient associations and social media precluded calculation of a response rate and likely introduced selection bias, potentially overestimating pain worsening and limiting generalizability. The cross-sectional nature precludes causal inference, and the lack of pre-IVF VAS scores prevents within-person comparisons. While current hormonal therapy at survey completion was recorded, detailed pre-IVF regimens were not systematically captured. Finally, the cohort’s overrepresentation of highly educated women may further limit generalizability.

### Future directions

Prospective longitudinal studies with event-anchored PROMs at key IVF milestones (before the stimulation, oocyte retrieval, embryo transfer, early luteal phase) should differentiate procedure-limited pain from persistent trajectories and identify high-risk patients early. More detailed exposure data on hormonal pretreatments, and post-IVF therapies—alongside psychological measures—are needed to clarify mechanisms and potential effect modifiers. Future trials should test supportive strategies initiated after early post-cycle flares (optimized analgesia, psychological care, individualized stimulation protocols), with outcomes including pain, quality of life, in addition to reproductive success.

## Conclusions

In this international cross-sectional survey, IVF was not pain-neutral for many women with endometriosis: nearly half reported worsening of pelvic pain. Immediate post-cycle flare identified those at highest risk of subsequent worsening, whereas live birth was associated with lower risk, indicating heterogeneous trajectories from procedure-limited pain to persistent flares. These hypothesis-generating findings support routine, event-anchored PROM monitoring across IVF steps and warrant prospective studies integrating hormonal exposures, psychological distress, and supportive care to test whether targeted supportive interventions reduce long-term pain while pursuing reproductive goals.

## Supplementary Material

hoag027_Supplementary_Data

## Data Availability

The full R scripts used for data cleaning and statistical analyses are publicly available in a dedicated GitHub repository: https://github.com/jonasvibert/fertipainR. Access to fully anonymized individual-level data can be granted upon reasonable request, subject to applicable ethical and data protection regulations.
