# Supplementary material for: IVF in endometriosis: emerging evidence of exacerbation of pelvic pain and potential predictors
Source: Hum Reprod Open. 2026 Mar 27;2026(2):hoag027. doi: 10.1093/hropen/hoag027 (PMC13091650; doi:10.1093/hropen/hoag027)
Supplement: hoag027_Supplementary_Data [file hoag027_supplementary_data.zip › Supplementary_File_S1._Questionnaire_instrument_(REDCap_export)_(1).pdf]

FERTIPAIN Questionnaire

In vitro fertilization pain experience in women with endometriosis (FERTIPAIN): a web survey

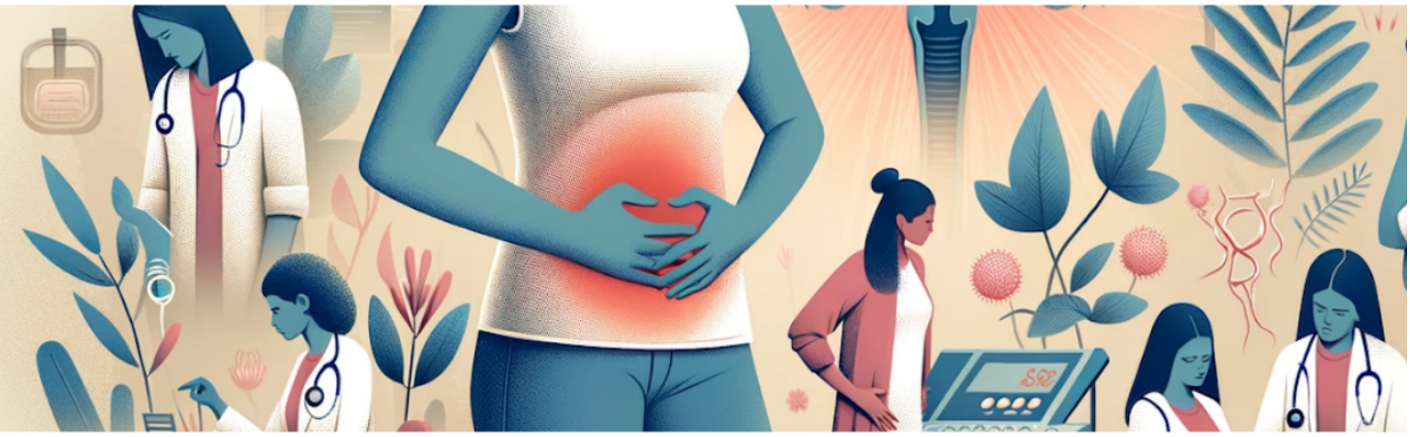

Dear patients,

Endometriosis is a complex medical condition and a common cause of infertility in women. This disease can make natural conception difficult, leading many women to turn to assisted reproductive treatments (ART), such as in vitro fertilization (IVF).

"Fertipain" is a research project conducted by the team at the fertility center of the University Hospital of Lausanne (Switzerland). This study aims to evaluate the pain experience during in vitro fertilization (IVF) among patients with endometriosis and to determine if the support provided by caregivers meets their specific needs.

Do you meet the following 2 criteria?

1) Diagnosis of endometriosis

2) Having undergone at least one cycle of IVF

Then participate in our study by completing the questionnaire below (approx. 10 min).

This questionnaire was evaluated using the tools provided by the Cantonal Ethics Commission for Research on Human Beings (CER-VD, Switzerland).

Anonymity is guaranteed.

For any questions or comments regarding this study, you can contact our team at the following address: endometriose@chuv.ch.

1 What age range do you fall into?

- ◯ 18-20 years
- ◯ 21-23 years
- ◯ 24-26 years
- ◯ 27-29 years
- ◯ 30-32 years
- ◯ 33-34 years
- ◯ 35-37 years
- ◯ 38-40 years
- ◯ 41-43 years
- ◯ 44-45 years
- ◯ 46-50 years
- ◯ 51-55 years
- ◯ 56-60 years
- ◯ 61-65 years
- ◯ 66-70 years
- ◯ 71-75 years
- ◯ 76-80 years
- ◯ 81-85 years
- ◯ 86-90 years
- ◯ >90 years

2 Which region were you born in?

- ◯ Europe
- ◯ Northern Africa
- ◯ Sub-Saharan Africa
- ◯ Middle-East
- ◯ Central Asia
- ◯ South-East Asia
- ◯ North America
- ◯ Central America
- ◯ South America
- ◯ Oceania
- ◯ Antartica

3 Which is your level of education?

- ◯ Primary school
- ◯ Secondary education (high school diploma, specialized diploma)
- ◯ Tertiary education (University, Technical University, University of Applied Sciences, higher professional education)

4 At what age were you diagnosed with endometriosis?

- ◯ < 15 years
- ◯ 15-20 years
- ◯ 21-25 years
- ◯ 26-30 years
- ◯ 31-35 years
- ◯ 36-40 years
- ◯ 41-45 years
- ◯ >45 years

5 Which was the time frame between symptoms onset and date of diagnosis ? (Months, if years please convert to months)

- ◯ 1
- ◯ 2
- ◯ 3
- ◯ 4
- ◯ 5
- ◯ 6
- ◯ 7
- ◯ 8
- ◯ 9
- ◯ 10
- ◯ 11
- ◯ 12 (1 year)
- ◯ 13
- ◯ 14
- ◯ 15
- ◯ 16
- ◯ 17
- ◯ 18
- ◯ 19
- ◯ 20
- ◯ 21
- ◯ 2
- ◯ 23
- ◯ 24 (2 years)
- ◯ 25
- ◯ 26
- ◯ 27
- ◯ 28
- ◯ 29
- ◯ 30
- ◯ 31
- ◯ 32
- ◯ 33
- ◯ 34
- ◯ 35
- ◯ 36 (3 years)
- ◯ 37
- ◯ 38
- ◯ 39
- ◯ 40
- ◯ 41
- ◯ 42
- ◯ 43
- ◯ 44
- ◯ 45
- ◯ 46
- ◯ 47
- ◯ 48 (4 years)
- ◯ 49
- ◯ 50
- ◯ 51
- ◯ 52
- ◯ 53
- ◯ 54
- ◯ 55
- ◯ 56
- ◯ 57
- ◯ 58
- ◯ 59
- ◯ 60 (5 years)
- ◯ 61
- ◯ 62
- ◯ 63
- ◯ 64
- ◯ 65
- ◯ 66
- ◯ 67
- ◯ 68
- ◯ 69
- ◯ 70

◯ 71
◯ 72 (6 years)
◯ 73
◯ 74
◯ 75
◯ 76
◯ 77
◯ 78
◯ 79
◯ 80
◯ 81
◯ 82
◯ 83
◯ 84 (7 years)
◯ 85
◯ 86
◯ 87
◯ 88
◯ 89
◯ 90
◯ 91
◯ 92
◯ 93
◯ 94
◯ 95
◯ 96 (8 years)
◯ 97
◯ 98
◯ 99
◯ 100
◯ 101
◯ 102
◯ 103
◯ 104
◯ 105
◯ 106
◯ 107
◯ 108 (9 years)
◯ 109
◯ 110
◯ 111
◯ 112
◯ 113
◯ 114
◯ 115
◯ 116
◯ 117
◯ 118
◯ 119
◯ 120 (10 years)
◯ 121
◯ 122
◯ 123
◯ 124
◯ 125
◯ 126
◯ 127
◯ 128
◯ 129
◯ 130
◯ 131
◯ 132 (11 years)
◯ >132 (11 years)
(months)

6	Have you ever had surgery for endometriosis before undergoing IVF?	◯ Yes<br>◯ No
6.1	Which lesion/s has/have been identified? (Check all that apply)	☐ Superficial endometriosis<br>☐ Endometrioma ("Chocolate ovarian cyst")<br>☐ Deep infiltrating endometriosis (i.e. bladder rectovaginal, ureteral,uterosacral ,diaphragmatic nodules)<br>☐ Adenomyosis ("endometriosis of the uterus")<br>☐ I don't remember / I don't know
6.2	Which disease stage has been assigned?	◯ ASRM I (minimal)<br>◯ ASRM II (mild)<br>◯ ASRM III (moderate)<br>◯ ASRM IV (severe)<br>◯ I don't remember / I don't know

7 How long did you try to conceive before undergoing your first IVF treatment? (Please provide the answer in months. If it has been several years, kindly convert it into months.)

- ◯ 1
- ◯ 2
- ◯ 3
- ◯ 4
- ◯ 5
- ◯ 6
- ◯ 7
- ◯ 8
- ◯ 9
- ◯ 10
- ◯ 11
- ◯ 12 (1 year)
- ◯ 13
- ◯ 14
- ◯ 15
- ◯ 16
- ◯ 17
- ◯ 18
- ◯ 19
- ◯ 20
- ◯ 21
- ◯ 22
- ◯ 23
- ◯ 24 (2 years)
- ◯ 25
- ◯ 26
- ◯ 27
- ◯ 28
- ◯ 29
- ◯ 30
- ◯ 31
- ◯ 32
- ◯ 33
- ◯ 34
- ◯ 35
- ◯ 36 (3 years)
- ◯ 37
- ◯ 38
- ◯ 39
- ◯ 40
- ◯ 41
- ◯ 42
- ◯ 43
- ◯ 44
- ◯ 45
- ◯ 46
- ◯ 47
- ◯ 48 (4 years)
- ◯ 49
- ◯ 50
- ◯ 51
- ◯ 52
- ◯ 53
- ◯ 54
- ◯ 55
- ◯ 56
- ◯ 57
- ◯ 58
- ◯ 59
- ◯ 60 (5 years)
- ◯ 61
- ◯ 62
- ◯ 63
- ◯ 64
- ◯ 65
- ◯ 66
- ◯ 67
- ◯ 68
- ◯ 69
- ◯ 70

◯ 71
◯ 72 (6 years)
◯ 73
◯ 74
◯ 75
◯ 76
◯ 77
◯ 78
◯ 79
◯ 80
◯ 81
◯ 82
◯ 83
◯ 84 (7 years)
◯ 85
◯ 86
◯ 87
◯ 88
◯ 89
◯ 90
◯ 91
◯ 92
◯ 93
◯ 94
◯ 95
◯ 96 (8 years)
◯ 97
◯ 98
◯ 99
◯ 100
◯ > 100
(months)

---

8 Did you give birth before IVF?

◯ Yes
◯ No

---

9 How many IVF "cycle" have you undergone? (A cycle starts by "the injection" and ends with pickups (oocyte retrieval)

◯ 1
◯ 2
◯ 3
◯ 4
◯ 5
◯ 6
◯ 7
◯ 8
◯ 9
◯ 10
◯ 11
◯ >11

Please answer the following questions regarding each IVF cycle.

1. cycle

9.1.1In which country did the procedure take place?

◯ Afghanistan
◯ Albania
◯ Algeria
◯ American Samoa
◯ Andorra
◯ Angola
◯ Anguilla
◯ Antarctica
◯ Antigua and Barbuda
◯ Argentina
◯ Armenia
◯ Aruba
◯ Australia
◯ Austria
◯ Azerbaijan
◯ Bahamas
◯ Bahrain
◯ Bangladesh
◯ Barbados
◯ Belarus
◯ Belgium
◯ Belize
◯ Benin
◯ Bermuda
◯ Bhutan
◯ Bolivia
◯ Bosnia and Herzegovina
◯ Botswana
◯ Brazil
◯ British Indian Ocean Territory
◯ British Virgin Islands
◯ Brunei Darussalam
◯ Bulgaria
◯ Burkina Faso
◯ Burundi
◯ Cape Verde
◯ Cambodia
◯ Cameroon
◯ Canada
◯ Cayman Islands
◯ Central African Republic
◯ Chad
◯ Chile
◯ China
◯ Christmas Island
◯ Cocos (Keeling) Islands
◯ Colombia
◯ Comoros
◯ Cook Islands
◯ Costa Rica
◯ Croatia
◯ Cuba
◯ Curaçao
◯ Cyprus
◯ Czech Republic
◯ Democratic Republic of the Congo
◯ Denmark
◯ Djibouti
◯ Dominica
◯ Dominican Republic
◯ East Timor
◯ Ecuador
◯ Egypt
◯ El Salvador
◯ Equatorial Guinea
◯ Eritrea
◯ Estonia
◯ Eswatini (Swaziland)
◯ Ethiopia
◯ Falkland Islands (Malvinas)

◯ Faroe Islands
◯ Fiji
◯ Finland
◯ France
◯ French Polynesia
◯ Gabon
◯ Gambia
◯ Georgia
◯ Germany
◯ Ghana
◯ Gibraltar
◯ Greece
◯ Greenland
◯ Grenada
◯ Guam
◯ Guatemala
◯ Guernsey
◯ Guinea
◯ Guinea-Bissau
◯ Guyana
◯ Haiti
◯ Honduras
◯ Hong Kong
◯ Hungary
◯ Iceland
◯ India
◯ Indonesia
◯ Iran
◯ Iraq
◯ Ireland
◯ Isle of Man
◯ Israel
◯ Italy
◯ Ivory Coast
◯ Jamaica
◯ Japan
◯ Jersey
◯ Jordan
◯ Kazakhstan
◯ Kenya
◯ Kiribati
◯ Kosovo
◯ Kuwait
◯ Kyrgyzstan
◯ Laos
◯ Latvia
◯ Lebanon
◯ Lesotho
◯ Liberia
◯ Libya
◯ Liechtenstein
◯ Lithuania
◯ Luxembourg
◯ Macau
◯ North Macedonia
◯ North Macedonia Madagascar
◯ Malawi
◯ Malaysia
◯ Maldives
◯ Mali
◯ Malta
◯ Marshall Islands
◯ Mauritania
◯ Mauritius
◯ Mayotte
◯ Mexico
◯ Micronesia
◯ Moldova
◯ Monaco
◯ Mongolia
◯ Montenegro

◯ Montserrat
◯ Morocco
◯ Mozambique
◯ Myanmar
◯ Namibia
◯ Nauru
◯ Nepal
◯ Netherlands
◯ Netherlands Antilles
◯ New Caledonia
◯ New Zealand
◯ Nicaragua
◯ Niger
◯ Nigeria
◯ Niue
◯ North Korea
◯ Northern Mariana Islands
◯ Norway
◯ Oman
◯ Pakistan
◯ Palau
◯ Palestine
◯ Panama
◯ Papua New Guinea
◯ Paraguay
◯ Peru
◯ Philippines
◯ Pitcairn
◯ Poland
◯ Portugal
◯ Puerto Rico
◯ Qatar
◯ Republic of the Congo
◯ Réunion
◯ Romania
◯ Russia
◯ Rwanda
◯ Saint Barthélemy
◯ Saint Helena
◯ Saint Kitts and Nevis
◯ Saint Lucia
◯ Saint Martin
◯ Saint Pierre and Miquelon
◯ Saint Vincent and the Grenadines
◯ Samoa
◯ San Marino
◯ São Tomé and Príncipe
◯ audi Arabia
◯ Senegal
◯ Serbia
◯ Seychelles
◯ Sierra Leone
◯ Singapore
◯ Sint Maarten
◯ Slovakia
◯ Slovenia
◯ Solomon Islands
◯ Somalia
◯ South Africa
◯ South Korea
◯ South Sudan
◯ Spain
◯ Sri Lanka
◯ Sudan
◯ Suriname
◯ Svalbard and Jan Mayen
◯ Sweden
◯ Switzerland
◯ Syria
◯ Taiwan
◯ Tajikistan

◯ Tanzania
◯ Thailand
◯ Togo
◯ Tokelau
◯ Tonga
◯ Trinidad and Tobago
◯ Tunisia
◯ Turkey
◯ Turkmenistan
◯ Turks and Caicos Islands
◯ Tuvalu
◯ United States Virgin Islands
◯ Uganda
◯ Ukraine
◯ United Arab Emirates
◯ United Kingdom
◯ United States of America
◯ Uruguay
◯ Uzbekistan
◯ Vanuatu
◯ Venezuela
◯ Vietnam
◯ Wallis and Futuna
◯ Western Sahara
◯ Yemen
◯ Zambia
◯ Zimbabwe
◯ Other

9.1.2 How long has it been since the procedure was performed?

◯ 0-2 years
◯ 3-4 years
◯ 5-6 years
◯ 7-8 years
◯ 9-10 years
◯ 11-12 years
◯ 13-14 years
◯ 15-16 years
◯ 17-18 years
◯ 19-20 years
◯ >20 years

9.1.3 At the time you underwent this in vitro fertilization (IVF), was there any evidence of an endometriotic lesion?

◯ Yes
◯ No
◯ I don't know / I don't remember anymore

9.1.4 Which lesion/s has/have been identified? (check all that apply)

☐ Superficial endometriosis
☐ Endometrioma ("Chocolate cyst of the ovary")
☐ Deep infiltrating endometriosis (i.e. bladder/rectovaginal/ureteral/uterosacral /diaphragmatic nodules)
☐ Adenomyosis ("endometriosis of the uterus")
☐ I don't remember / I don't know

9.1.5 Where was it performed?

◯ Public institution
◯ Private institution

9.1.6 Did you have to spend money without being refunded by health insurance?

◯ Yes
◯ No

9.1.7 How many embryos did you obtain?

- ◯ 0
- ◯ 1
- ◯ 2
- ◯ 3
- ◯ 4
- ◯ 5
- ◯ 6
- ◯ 7
- ◯ 8
- ◯ 9
- ◯ 10
- ◯ 11
- ◯ 12
- ◯ >12
- ◯ I don't know anymore

9.1.8 How many embryo-transfer did you perform?

- ◯ 0
- ◯ 1
- ◯ 2
- ◯ 3
- ◯ 4
- ◯ 5
- ◯ 6
- ◯ 7
- ◯ 8
- ◯ 9
- ◯ 10
- ◯ 11
- ◯ 12
- ◯ >12
- ◯ I don't know anymore

9.1.9 Did you get pregnant?

- ◯ Yes
- ◯ No

9.1.10 Did you deliver at the end of this pregnancy?

- ◯ Yes
- ◯ No

9.1.11 Have you had any complications related to endometriosis during IVF or during pregnancy requiring hospitalization?

- ◯ Yes
- ◯ No

9.1.12 Please check all that apply (Multiple answers possible):

- ☐ Blood in abdomen (hemoperitoneum)
- ☐ Acute bowel obstruction (ileus)
- ☐ Unbearable pain
- ☐ Other complication
- ☐ I don't remember

9.1.13 During and right after this IVF cycle, how would you describe (if any) the change in endometriosis pain?

- ◯ Significantly improved
- ◯ Improved
- ◯ Minimally improved
- ◯ No change
- ◯ Minimally worse
- ◯ Worse
- ◯ Significantly worse

2th cycle

9.2.1In which country did the procedure take place?

◯ Afghanistan
◯ Albania
◯ Algeria
◯ American Samoa
◯ Andorra
◯ Angola
◯ Anguilla
◯ Antarctica
◯ Antigua and Barbuda
◯ Argentina
◯ Armenia
◯ Aruba
◯ Australia
◯ Austria
◯ Azerbaijan
◯ Bahamas
◯ Bahrain
◯ Bangladesh
◯ Barbados
◯ Belarus
◯ Belgium
◯ Belize
◯ Benin
◯ Bermuda
◯ Bhutan
◯ Bolivia
◯ Bosnia and Herzegovina
◯ Botswana
◯ Brazil
◯ British Indian Ocean Territory
◯ British Virgin Islands
◯ Brunei Darussalam
◯ Bulgaria
◯ Burkina Faso
◯ Burundi
◯ Cape Verde
◯ Cambodia
◯ Cameroon
◯ Canada
◯ Cayman Islands
◯ Central African Republic
◯ Chad
◯ Chile
◯ China
◯ Christmas Island
◯ Cocos (Keeling) Islands
◯ Colombia
◯ Comoros
◯ Cook Islands
◯ Costa Rica
◯ Croatia
◯ Cuba
◯ Curaçao
◯ Cyprus
◯ Czech Republic
◯ Democratic Republic of the Congo
◯ Denmark
◯ Djibouti
◯ Dominica
◯ Dominican Republic
◯ East Timor
◯ Ecuador
◯ Egypt
◯ El Salvador
◯ Equatorial Guinea
◯ Eritrea
◯ Estonia
◯ Eswatini (Swaziland)
◯ Ethiopia
◯ Falkland Islands (Malvinas)

◯ Faroe Islands
◯ Fiji
◯ Finland
◯ France
◯ French Polynesia
◯ Gabon
◯ Gambia
◯ Georgia
◯ Germany
◯ Ghana
◯ Gibraltar
◯ Greece
◯ Greenland
◯ Grenada
◯ Guam
◯ Guatemala
◯ Guernsey
◯ Guinea
◯ Guinea-Bissau
◯ Guyana
◯ Haiti
◯ Honduras
◯ Hong Kong
◯ Hungary
◯ Iceland
◯ India
◯ Indonesia
◯ Iran
◯ Iraq
◯ Ireland
◯ Isle of Man
◯ Israel
◯ Italy
◯ Ivory Coast
◯ Jamaica
◯ Japan
◯ Jersey
◯ Jordan
◯ Kazakhstan
◯ Kenya
◯ Kiribati
◯ Kosovo
◯ Kuwait
◯ Kyrgyzstan
◯ Laos
◯ Latvia
◯ Lebanon
◯ Lesotho
◯ Liberia
◯ Libya
◯ Liechtenstein
◯ Lithuania
◯ Luxembourg
◯ Macau
◯ North Macedonia
◯ North Macedonia Madagascar
◯ Malawi
◯ Malaysia
◯ Maldives
◯ Mali
◯ Malta
◯ Marshall Islands
◯ Mauritania
◯ Mauritius
◯ Mayotte
◯ Mexico
◯ Micronesia
◯ Moldova
◯ Monaco
◯ Mongolia
◯ Montenegro

◯ Montserrat
◯ Morocco
◯ Mozambique
◯ Myanmar
◯ Namibia
◯ Nauru
◯ Nepal
◯ Netherlands
◯ Netherlands Antilles
◯ New Caledonia
◯ New Zealand
◯ Nicaragua
◯ Niger
◯ Nigeria
◯ Niue
◯ North Korea
◯ Northern Mariana Islands
◯ Norway
◯ Oman
◯ Pakistan
◯ Palau
◯ Palestine
◯ Panama
◯ Papua New Guinea
◯ Paraguay
◯ Peru
◯ Philippines
◯ Pitcairn
◯ Poland
◯ Portugal
◯ Puerto Rico
◯ Qatar
◯ Republic of the Congo
◯ Réunion
◯ Romania
◯ Russia
◯ Rwanda
◯ Saint Barthélemy
◯ Saint Helena
◯ Saint Kitts and Nevis
◯ Saint Lucia
◯ Saint Martin
◯ Saint Pierre and Miquelon
◯ Saint Vincent and the Grenadines
◯ Samoa
◯ San Marino
◯ São Tomé and Príncipe
◯ audi Arabia
◯ Senegal
◯ Serbia
◯ Seychelles
◯ Sierra Leone
◯ Singapore
◯ Sint Maarten
◯ Slovakia
◯ Slovenia
◯ Solomon Islands
◯ Somalia
◯ South Africa
◯ South Korea
◯ South Sudan
◯ Spain
◯ Sri Lanka
◯ Sudan
◯ Suriname
◯ Svalbard and Jan Mayen
◯ Sweden
◯ Switzerland
◯ Syria
◯ Taiwan
◯ Tajikistan

◯ Tanzania
◯ Thailand
◯ Togo
◯ Tokelau
◯ Tonga
◯ Trinidad and Tobago
◯ Tunisia
◯ Turkey
◯ Turkmenistan
◯ Turks and Caicos Islands
◯ Tuvalu
◯ United States Virgin Islands
◯ Uganda
◯ Ukraine
◯ United Arab Emirates
◯ United Kingdom
◯ United States of America
◯ Uruguay
◯ Uzbekistan
◯ Vanuatu
◯ Venezuela
◯ Vietnam
◯ Wallis and Futuna
◯ Western Sahara
◯ Yemen
◯ Zambia
◯ Zimbabwe
◯ Other

9.2.2 How long has it been since the procedure was performed?

◯ 0-2 years
◯ 3-4 years
◯ 5-6 years
◯ 7-8 years
◯ 9-10 years
◯ 11-12 years
◯ 13-14 years
◯ 15-16 years
◯ 17-18 years
◯ 19-20 years
◯ >20 years

9.2.3 At the time you underwent this in vitro fertilization (IVF), was there any evidence of an endometriotic lesion?

◯ Yes
◯ No
◯ I don't know / I don't remember anymore

9.2.4 Which lesion/s has/have been identified? (check all that apply)

☐ Superficial endometriosis
☐ Endometrioma ("Chocolate cyst of the ovary")
☐ Deep infiltrating endometriosis (i.e. bladder/rectovaginal/ureteral/uterosacral /diaphragmatic nodules)
☐ Adenomyosis ("endometriosis of the uterus")
☐ I don't remember / I don't know

9.2.5 Where was the procedure performed?

◯ Public institution
◯ Private institution

9.2.6 Did you have to spend money without being refunded by health insurance?

◯ Yes
◯ No

9.2.7 How many embryos did you obtain?

- ◯ 0
- ◯ 1
- ◯ 2
- ◯ 3
- ◯ 4
- ◯ 5
- ◯ 6
- ◯ 7
- ◯ 8
- ◯ 9
- ◯ 10
- ◯ 11
- ◯ 12
- ◯ >12
- ◯ I don't know anymore

9.2.8 How many embryo-transfer did you perform?

- ◯ 0
- ◯ 1
- ◯ 2
- ◯ 3
- ◯ 4
- ◯ 5
- ◯ 6
- ◯ 7
- ◯ 8
- ◯ 9
- ◯ 10
- ◯ 11
- ◯ 12
- ◯ >12
- ◯ I don't know anymore

9.2.9 Did you get pregnant?

- ◯ Yes
- ◯ No

9.2.10 Did you deliver at the end of this pregnancy?

- ◯ Yes
- ◯ No

9.2.11 Have you had any complications related to endometriosis during IVF or during pregnancy requiring hospitalization?

- ◯ Yes
- ◯ No

9.2.12 Please check all that apply (Multiple answers possible):

- ☐ Blood in abdomen (hemoperitoneum)
- ☐ Acute bowel obstruction (ileus)
- ☐ Unbearable pain
- ☐ Other complication
- ☐ I don't remember

9.2.13 During and right after this IVF cycle, how would you describe (if any) the change in endometriosis pain?

- ◯ Significantly improved
- ◯ Improved
- ◯ Minimally improved
- ◯ No change
- ◯ Minimally worse
- ◯ Worse
- ◯ Significantly worse

3th cycle

9.3.1 In which country did the procedure take place?

- ◯ Afghanistan
- ◯ Albania
- ◯ Algeria
- ◯ American Samoa
- ◯ Andorra
- ◯ Angola
- ◯ Anguilla
- ◯ Antarctica
- ◯ Antigua and Barbuda
- ◯ Argentina
- ◯ Armenia
- ◯ Aruba
- ◯ Australia
- ◯ Austria
- ◯ Azerbaijan
- ◯ Bahamas
- ◯ Bahrain
- ◯ Bangladesh
- ◯ Barbados
- ◯ Belarus
- ◯ Belgium
- ◯ Belize
- ◯ Benin
- ◯ Bermuda
- ◯ Bhutan
- ◯ Bolivia
- ◯ Bosnia and Herzegovina
- ◯ Botswana
- ◯ Brazil
- ◯ British Indian Ocean Territory
- ◯ British Virgin Islands
- ◯ Brunei Darussalam
- ◯ Bulgaria
- ◯ Burkina Faso
- ◯ Burundi
- ◯ Cape Verde
- ◯ Cambodia
- ◯ Cameroon
- ◯ Canada
- ◯ Cayman Islands
- ◯ Central African Republic
- ◯ Chad
- ◯ Chile
- ◯ China
- ◯ Christmas Island
- ◯ Cocos (Keeling) Islands
- ◯ Colombia
- ◯ Comoros
- ◯ Cook Islands
- ◯ Costa Rica
- ◯ Croatia
- ◯ Cuba
- ◯ Curaçao
- ◯ Cyprus
- ◯ Czech Republic
- ◯ Democratic Republic of the Congo
- ◯ Denmark
- ◯ Djibouti
- ◯ Dominica
- ◯ Dominican Republic
- ◯ East Timor
- ◯ Ecuador
- ◯ Egypt
- ◯ El Salvador
- ◯ Equatorial Guinea
- ◯ Eritrea
- ◯ Estonia
- ◯ Eswatini (Swaziland)
- ◯ Ethiopia
- ◯ Falkland Islands (Malvinas)

◯ Faroe Islands
◯ Fiji
◯ Finland
◯ France
◯ French Polynesia
◯ Gabon
◯ Gambia
◯ Georgia
◯ Germany
◯ Ghana
◯ Gibraltar
◯ Greece
◯ Greenland
◯ Grenada
◯ Guam
◯ Guatemala
◯ Guernsey
◯ Guinea
◯ Guinea-Bissau
◯ Guyana
◯ Haiti
◯ Honduras
◯ Hong Kong
◯ Hungary
◯ Iceland
◯ India
◯ Indonesia
◯ Iran
◯ Iraq
◯ Ireland
◯ Isle of Man
◯ Israel
◯ Italy
◯ Ivory Coast
◯ Jamaica
◯ Japan
◯ Jersey
◯ Jordan
◯ Kazakhstan
◯ Kenya
◯ Kiribati
◯ Kosovo
◯ Kuwait
◯ Kyrgyzstan
◯ Laos
◯ Latvia
◯ Lebanon
◯ Lesotho
◯ Liberia
◯ Libya
◯ Liechtenstein
◯ Lithuania
◯ Luxembourg
◯ Macau
◯ North Macedonia
◯ North Macedonia Madagascar
◯ Malawi
◯ Malaysia
◯ Maldives
◯ Mali
◯ Malta
◯ Marshall Islands
◯ Mauritania
◯ Mauritius
◯ Mayotte
◯ Mexico
◯ Micronesia
◯ Moldova
◯ Monaco
◯ Mongolia
◯ Montenegro

◯ Montserrat
◯ Morocco
◯ Mozambique
◯ Myanmar
◯ Namibia
◯ Nauru
◯ Nepal
◯ Netherlands
◯ Netherlands Antilles
◯ New Caledonia
◯ New Zealand
◯ Nicaragua
◯ Niger
◯ Nigeria
◯ Niue
◯ North Korea
◯ Northern Mariana Islands
◯ Norway
◯ Oman
◯ Pakistan
◯ Palau
◯ Palestine
◯ Panama
◯ Papua New Guinea
◯ Paraguay
◯ Peru
◯ Philippines
◯ Pitcairn
◯ Poland
◯ Portugal
◯ Puerto Rico
◯ Qatar
◯ Republic of the Congo
◯ Réunion
◯ Romania
◯ Russia
◯ Rwanda
◯ Saint Barthélemy
◯ Saint Helena
◯ Saint Kitts and Nevis
◯ Saint Lucia
◯ Saint Martin
◯ Saint Pierre and Miquelon
◯ Saint Vincent and the Grenadines
◯ Samoa
◯ San Marino
◯ São Tomé and Príncipe
◯ audi Arabia
◯ Senegal
◯ Serbia
◯ Seychelles
◯ Sierra Leone
◯ Singapore
◯ Sint Maarten
◯ Slovakia
◯ Slovenia
◯ Solomon Islands
◯ Somalia
◯ South Africa
◯ South Korea
◯ South Sudan
◯ Spain
◯ Sri Lanka
◯ Sudan
◯ Suriname
◯ Svalbard and Jan Mayen
◯ Sweden
◯ Switzerland
◯ Syria
◯ Taiwan
◯ Tajikistan

- ◯ Tanzania
- ◯ Thailand
- ◯ Togo
- ◯ Tokelau
- ◯ Tonga
- ◯ Trinidad and Tobago
- ◯ Tunisia
- ◯ Turkey
- ◯ Turkmenistan
- ◯ Turks and Caicos Islands
- ◯ Tuvalu
- ◯ United States Virgin Islands
- ◯ Uganda
- ◯ Ukraine
- ◯ United Arab Emirates
- ◯ United Kingdom
- ◯ United States of America
- ◯ Uruguay
- ◯ Uzbekistan
- ◯ Vanuatu
- ◯ Venezuela
- ◯ Vietnam
- ◯ Wallis and Futuna
- ◯ Western Sahara
- ◯ Yemen
- ◯ Zambia
- ◯ Zimbabwe
- ◯ Other

9.3.2 How long has it been since the procedure was performed?

- ◯ 0-2 years
- ◯ 3-4 years
- ◯ 5-6 years
- ◯ 7-8 years
- ◯ 9-10 years
- ◯ 11-12 years
- ◯ 13-14 years
- ◯ 15-16 years
- ◯ 17-18 years
- ◯ 19-20 years
- ◯ >20 years

9.3.3 At the time you underwent this in vitro fertilization (IVF), was there any evidence of an endometriotic lesion?

- ◯ Yes
- ◯ No
- ◯ I don't know / I don't remember anymore

9.3.4 Which lesion/s has/have been identified? (check all that apply)

- ☐ Superficial endometriosis
- ☐ Endometrioma ("Chocolate cyst of the ovary")
- ☐ Deep infiltrating endometriosis (i.e. bladder/rectovaginal/ureteral/uterosacral /diaphragmatic nodules)
- ☐ Adenomyosis ("endometriosis of the uterus")
- ☐ I don't remember / I don't know

9.3.5 Where was the procedure performed?

- ◯ Public institution
- ◯ Private institution

9.3.6 Did you have to spend money without being refunded by health insurance?

- ◯ Yes
- ◯ No

9.3.7 How many embryos did you obtain?

- ◯ 0
- ◯ 1
- ◯ 2
- ◯ 3
- ◯ 4
- ◯ 5
- ◯ 6
- ◯ 7
- ◯ 8
- ◯ 9
- ◯ 10
- ◯ 11
- ◯ 12
- ◯ >12
- ◯ I don't know anymore

9.3.8 How many embryo-transfer did you perform?

- ◯ 0
- ◯ 1
- ◯ 2
- ◯ 3
- ◯ 4
- ◯ 5
- ◯ 6
- ◯ 7
- ◯ 8
- ◯ 9
- ◯ 10
- ◯ 11
- ◯ 12
- ◯ >12
- ◯ I don't know anymore

9.3.9 Did you get pregnant?

- ◯ Yes
- ◯ No

9.3.10 Did you deliver at the end of this pregnancy?

- ◯ Yes
- ◯ No

9.3.11 Have you had any complications related to endometriosis during IVF or during pregnancy requiring hospitalization?

- ◯ Yes
- ◯ No

9.3.12 Please check all that apply (Multiple answers possible):

- ☐ Blood in abdomen (hemoperitoneum)
- ☐ Acute bowel obstruction (ileus)
- ☐ Unbearable pain
- ☐ Other complication
- ☐ I don't remember

9.3.13 During and right after this IVF cycle, how would you describe (if any) the change in endometriosis pain?

- ◯ Significantly improved
- ◯ Improved
- ◯ Minimally improved
- ◯ No change
- ◯ Minimally worse
- ◯ Worse
- ◯ Significantly worse

4th cycle

9.4.1In which country did the procedure take place?

◯ Afghanistan
◯ Albania
◯ Algeria
◯ American Samoa
◯ Andorra
◯ Angola
◯ Anguilla
◯ Antarctica
◯ Antigua and Barbuda
◯ Argentina
◯ Armenia
◯ Aruba
◯ Australia
◯ Austria
◯ Azerbaijan
◯ Bahamas
◯ Bahrain
◯ Bangladesh
◯ Barbados
◯ Belarus
◯ Belgium
◯ Belize
◯ Benin
◯ Bermuda
◯ Bhutan
◯ Bolivia
◯ Bosnia and Herzegovina
◯ Botswana
◯ Brazil
◯ British Indian Ocean Territory
◯ British Virgin Islands
◯ Brunei Darussalam
◯ Bulgaria
◯ Burkina Faso
◯ Burundi
◯ Cape Verde
◯ Cambodia
◯ Cameroon
◯ Canada
◯ Cayman Islands
◯ Central African Republic
◯ Chad
◯ Chile
◯ China
◯ Christmas Island
◯ Cocos (Keeling) Islands
◯ Colombia
◯ Comoros
◯ Cook Islands
◯ Costa Rica
◯ Croatia
◯ Cuba
◯ Curaçao
◯ Cyprus
◯ Czech Republic
◯ Democratic Republic of the Congo
◯ Denmark
◯ Djibouti
◯ Dominica
◯ Dominican Republic
◯ East Timor
◯ Ecuador
◯ Egypt
◯ El Salvador
◯ Equatorial Guinea
◯ Eritrea
◯ Estonia
◯ Eswatini (Swaziland)
◯ Ethiopia
◯ Falkland Islands (Malvinas)

◯ Faroe Islands
◯ Fiji
◯ Finland
◯ France
◯ French Polynesia
◯ Gabon
◯ Gambia
◯ Georgia
◯ Germany
◯ Ghana
◯ Gibraltar
◯ Greece
◯ Greenland
◯ Grenada
◯ Guam
◯ Guatemala
◯ Guernsey
◯ Guinea
◯ Guinea-Bissau
◯ Guyana
◯ Haiti
◯ Honduras
◯ Hong Kong
◯ Hungary
◯ Iceland
◯ India
◯ Indonesia
◯ Iran
◯ Iraq
◯ Ireland
◯ Isle of Man
◯ Israel
◯ Italy
◯ Ivory Coast
◯ Jamaica
◯ Japan
◯ Jersey
◯ Jordan
◯ Kazakhstan
◯ Kenya
◯ Kiribati
◯ Kosovo
◯ Kuwait
◯ Kyrgyzstan
◯ Laos
◯ Latvia
◯ Lebanon
◯ Lesotho
◯ Liberia
◯ Libya
◯ Liechtenstein
◯ Lithuania
◯ Luxembourg
◯ Macau
◯ North Macedonia
◯ North Macedonia Madagascar
◯ Malawi
◯ Malaysia
◯ Maldives
◯ Mali
◯ Malta
◯ Marshall Islands
◯ Mauritania
◯ Mauritius
◯ Mayotte
◯ Mexico
◯ Micronesia
◯ Moldova
◯ Monaco
◯ Mongolia
◯ Montenegro

◯ Montserrat
◯ Morocco
◯ Mozambique
◯ Myanmar
◯ Namibia
◯ Nauru
◯ Nepal
◯ Netherlands
◯ Netherlands Antilles
◯ New Caledonia
◯ New Zealand
◯ Nicaragua
◯ Niger
◯ Nigeria
◯ Niue
◯ North Korea
◯ Northern Mariana Islands
◯ Norway
◯ Oman
◯ Pakistan
◯ Palau
◯ Palestine
◯ Panama
◯ Papua New Guinea
◯ Paraguay
◯ Peru
◯ Philippines
◯ Pitcairn
◯ Poland
◯ Portugal
◯ Puerto Rico
◯ Qatar
◯ Republic of the Congo
◯ Réunion
◯ Romania
◯ Russia
◯ Rwanda
◯ Saint Barthélemy
◯ Saint Helena
◯ Saint Kitts and Nevis
◯ Saint Lucia
◯ Saint Martin
◯ Saint Pierre and Miquelon
◯ Saint Vincent and the Grenadines
◯ Samoa
◯ San Marino
◯ São Tomé and Príncipe
◯ audi Arabia
◯ Senegal
◯ Serbia
◯ Seychelles
◯ Sierra Leone
◯ Singapore
◯ Sint Maarten
◯ Slovakia
◯ Slovenia
◯ Solomon Islands
◯ Somalia
◯ South Africa
◯ South Korea
◯ South Sudan
◯ Spain
◯ Sri Lanka
◯ Sudan
◯ Suriname
◯ Svalbard and Jan Mayen
◯ Sweden
◯ Switzerland
◯ Syria
◯ Taiwan
◯ Tajikistan

◯ Tanzania
◯ Thailand
◯ Togo
◯ Tokelau
◯ Tonga
◯ Trinidad and Tobago
◯ Tunisia
◯ Turkey
◯ Turkmenistan
◯ Turks and Caicos Islands
◯ Tuvalu
◯ United States Virgin Islands
◯ Uganda
◯ Ukraine
◯ United Arab Emirates
◯ United Kingdom
◯ United States of America
◯ Uruguay
◯ Uzbekistan
◯ Vanuatu
◯ Venezuela
◯ Vietnam
◯ Wallis and Futuna
◯ Western Sahara
◯ Yemen
◯ Zambia
◯ Zimbabwe
◯ Other

9.4.2 How long has it been since the procedure was performed?

◯ 0-2 years
◯ 3-4 years
◯ 5-6 years
◯ 7-8 years
◯ 9-10 years
◯ 11-12 years
◯ 13-14 years
◯ 15-16 years
◯ 17-18 years
◯ 19-20 years
◯ >20 years

9.4.3 At the time you underwent this in vitro fertilization (IVF), was there any evidence of an endometriotic lesion?

◯ Yes
◯ No
◯ I don't know / I don't remember anymore

9.4.4 Which lesion/s has/have been identified? (check all that apply)

☐ Superficial endometriosis
☐ Endometrioma ("Chocolate cyst of the ovary")
☐ Deep infiltrating endometriosis (i.e. bladder/rectovaginal/ureteral/uterosacral /diaphragmatic nodules)
☐ Adenomyosis ("endometriosis of the uterus")
☐ I don't remember / I don't know

9.4.5 Where was the procedure performed?

◯ Public institution
◯ Private institution

9.4.6 Did you have to spend money without being refunded by health insurance?

◯ Yes
◯ No

9.4.7 How many embryos did you obtain?

- ◯ 0
- ◯ 1
- ◯ 2
- ◯ 3
- ◯ 4
- ◯ 5
- ◯ 6
- ◯ 7
- ◯ 8
- ◯ 9
- ◯ 10
- ◯ 11
- ◯ 12
- ◯ >12
- ◯ I don't know anymore

9.4.8 How many embryo-transfer did you perform?

- ◯ 0
- ◯ 1
- ◯ 2
- ◯ 3
- ◯ 4
- ◯ 5
- ◯ 6
- ◯ 7
- ◯ 8
- ◯ 9
- ◯ 10
- ◯ 11
- ◯ 12
- ◯ >12
- ◯ I don't know anymore

9.4.9 Did you get pregnant?

- ◯ Yes
- ◯ No

9.4.10 Did you deliver at the end of this pregnancy?

- ◯ Yes
- ◯ No

9.4.11 Have you had any complications related to endometriosis during IVF or during pregnancy requiring hospitalization?

- ◯ Yes
- ◯ No

9.4.12 Please check all that apply (Multiple answers possible):

- ☐ Blood in abdomen (hemoperitoneum)
- ☐ Acute bowel obstruction (ileus)
- ☐ Unbearable pain
- ☐ Other complication
- ☐ I don't remember

9.4.13 During and right after this IVF cycle, how would you describe (if any) the change in endometriosis pain?

- ◯ Significantly improved
- ◯ Improved
- ◯ Minimally improved
- ◯ No change
- ◯ Minimally worse
- ◯ Worse
- ◯ Significantly worse

5th cycle

9.5.1 In which country did the procedure take place?

- ◯ Afghanistan
- ◯ Albania
- ◯ Algeria
- ◯ American Samoa
- ◯ Andorra
- ◯ Angola
- ◯ Anguilla
- ◯ Antarctica
- ◯ Antigua and Barbuda
- ◯ Argentina
- ◯ Armenia
- ◯ Aruba
- ◯ Australia
- ◯ Austria
- ◯ Azerbaijan
- ◯ Bahamas
- ◯ Bahrain
- ◯ Bangladesh
- ◯ Barbados
- ◯ Belarus
- ◯ Belgium
- ◯ Belize
- ◯ Benin
- ◯ Bermuda
- ◯ Bhutan
- ◯ Bolivia
- ◯ Bosnia and Herzegovina
- ◯ Botswana
- ◯ Brazil
- ◯ British Indian Ocean Territory
- ◯ British Virgin Islands
- ◯ Brunei Darussalam
- ◯ Bulgaria
- ◯ Burkina Faso
- ◯ Burundi
- ◯ Cape Verde
- ◯ Cambodia
- ◯ Cameroon
- ◯ Canada
- ◯ Cayman Islands
- ◯ Central African Republic
- ◯ Chad
- ◯ Chile
- ◯ China
- ◯ Christmas Island
- ◯ Cocos (Keeling) Islands
- ◯ Colombia
- ◯ Comoros
- ◯ Cook Islands
- ◯ Costa Rica
- ◯ Croatia
- ◯ Cuba
- ◯ Curaçao
- ◯ Cyprus
- ◯ Czech Republic
- ◯ Democratic Republic of the Congo
- ◯ Denmark
- ◯ Djibouti
- ◯ Dominica
- ◯ Dominican Republic
- ◯ East Timor
- ◯ Ecuador
- ◯ Egypt
- ◯ El Salvador
- ◯ Equatorial Guinea
- ◯ Eritrea
- ◯ Estonia
- ◯ Eswatini (Swaziland)
- ◯ Ethiopia
- ◯ Falkland Islands (Malvinas)

◯ Faroe Islands
◯ Fiji
◯ Finland
◯ France
◯ French Polynesia
◯ Gabon
◯ Gambia
◯ Georgia
◯ Germany
◯ Ghana
◯ Gibraltar
◯ Greece
◯ Greenland
◯ Grenada
◯ Guam
◯ Guatemala
◯ Guernsey
◯ Guinea
◯ Guinea-Bissau
◯ Guyana
◯ Haiti
◯ Honduras
◯ Hong Kong
◯ Hungary
◯ Iceland
◯ India
◯ Indonesia
◯ Iran
◯ Iraq
◯ Ireland
◯ Isle of Man
◯ Israel
◯ Italy
◯ Ivory Coast
◯ Jamaica
◯ Japan
◯ Jersey
◯ Jordan
◯ Kazakhstan
◯ Kenya
◯ Kiribati
◯ Kosovo
◯ Kuwait
◯ Kyrgyzstan
◯ Laos
◯ Latvia
◯ Lebanon
◯ Lesotho
◯ Liberia
◯ Libya
◯ Liechtenstein
◯ Lithuania
◯ Luxembourg
◯ Macau
◯ North Macedonia
◯ North Macedonia Madagascar
◯ Malawi
◯ Malaysia
◯ Maldives
◯ Mali
◯ Malta
◯ Marshall Islands
◯ Mauritania
◯ Mauritius
◯ Mayotte
◯ Mexico
◯ Micronesia
◯ Moldova
◯ Monaco
◯ Mongolia
◯ Montenegro

◯ Montserrat
◯ Morocco
◯ Mozambique
◯ Myanmar
◯ Namibia
◯ Nauru
◯ Nepal
◯ Netherlands
◯ Netherlands Antilles
◯ New Caledonia
◯ New Zealand
◯ Nicaragua
◯ Niger
◯ Nigeria
◯ Niue
◯ North Korea
◯ Northern Mariana Islands
◯ Norway
◯ Oman
◯ Pakistan
◯ Palau
◯ Palestine
◯ Panama
◯ Papua New Guinea
◯ Paraguay
◯ Peru
◯ Philippines
◯ Pitcairn
◯ Poland
◯ Portugal
◯ Puerto Rico
◯ Qatar
◯ Republic of the Congo
◯ Réunion
◯ Romania
◯ Russia
◯ Rwanda
◯ Saint Barthélemy
◯ Saint Helena
◯ Saint Kitts and Nevis
◯ Saint Lucia
◯ Saint Martin
◯ Saint Pierre and Miquelon
◯ Saint Vincent and the Grenadines
◯ Samoa
◯ San Marino
◯ São Tomé and Príncipe
◯ audi Arabia
◯ Senegal
◯ Serbia
◯ Seychelles
◯ Sierra Leone
◯ Singapore
◯ Sint Maarten
◯ Slovakia
◯ Slovenia
◯ Solomon Islands
◯ Somalia
◯ South Africa
◯ South Korea
◯ South Sudan
◯ Spain
◯ Sri Lanka
◯ Sudan
◯ Suriname
◯ Svalbard and Jan Mayen
◯ Sweden
◯ Switzerland
◯ Syria
◯ Taiwan
◯ Tajikistan

- ◯ Tanzania
- ◯ Thailand
- ◯ Togo
- ◯ Tokelau
- ◯ Tonga
- ◯ Trinidad and Tobago
- ◯ Tunisia
- ◯ Turkey
- ◯ Turkmenistan
- ◯ Turks and Caicos Islands
- ◯ Tuvalu
- ◯ United States Virgin Islands
- ◯ Uganda
- ◯ Ukraine
- ◯ United Arab Emirates
- ◯ United Kingdom
- ◯ United States of America
- ◯ Uruguay
- ◯ Uzbekistan
- ◯ Vanuatu
- ◯ Venezuela
- ◯ Vietnam
- ◯ Wallis and Futuna
- ◯ Western Sahara
- ◯ Yemen
- ◯ Zambia
- ◯ Zimbabwe
- ◯ Other

9.5.2 How long has it been since the procedure was performed?

- ◯ 0-2 years
- ◯ 3-4 years
- ◯ 5-6 years
- ◯ 7-8 years
- ◯ 9-10 years
- ◯ 11-12 years
- ◯ 13-14 years
- ◯ 15-16 years
- ◯ 17-18 years
- ◯ 19-20 years
- ◯ >20 years

9.5.3 At the time you underwent this in vitro fertilization (IVF), was there any evidence of an endometriotic lesion?

- ◯ Yes
- ◯ No
- ◯ I don't know / I don't remember anymore

9.5.4 Which lesion/s has/have been identified? (check all that apply)

- ☐ Superficial endometriosis
- ☐ Endometrioma ("Chocolate cyst of the ovary")
- ☐ Deep infiltrating endometriosis (i.e. bladder/rectovaginal/ureteral/uterosacral /diaphragmatic nodules)
- ☐ Adenomyosis ("endometriosis of the uterus")
- ☐ I don't remember / I don't know

9.5.5 Where was the procedure performed?

- ◯ Public institution
- ◯ Private institution

9.5.6 Did you have to spend money without being refunded by health insurance?

- ◯ Yes
- ◯ No

9.5.7 How many embryos did you obtain?

- ◯ 0
- ◯ 1
- ◯ 2
- ◯ 3
- ◯ 4
- ◯ 5
- ◯ 6
- ◯ 7
- ◯ 8
- ◯ 9
- ◯ 10
- ◯ 11
- ◯ 12
- ◯ >12
- ◯ I don't know anymore

9.5.8 How many embryo-transfer did you perform?

- ◯ 0
- ◯ 1
- ◯ 2
- ◯ 3
- ◯ 4
- ◯ 5
- ◯ 6
- ◯ 7
- ◯ 8
- ◯ 9
- ◯ 10
- ◯ 11
- ◯ 12
- ◯ >12
- ◯ I don't know anymore

9.5.9 Did you get pregnant?

- ◯ Yes
- ◯ No

9.5.10 Did you deliver at the end of this pregnancy?

- ◯ Yes
- ◯ No

9.5.11 Have you had any complications related to endometriosis during IVF or during pregnancy requiring hospitalization?

- ◯ Yes
- ◯ No

9.5.12 Please check all that apply (Multiple answers possible):

- ☐ Blood in abdomen (hemoperitoneum)
- ☐ Acute bowel obstruction (ileus)
- ☐ Unbearable pain
- ☐ Other complication
- ☐ I don't remember

9.5.13 During and right after this IVF cycle, how would you describe (if any) the change in endometriosis pain?

- ◯ Significantly improved
- ◯ Improved
- ◯ Minimally improved
- ◯ No change
- ◯ Minimally worse
- ◯ Worse
- ◯ Significantly worse

6th cycle

9.6.1In which country did the procedure take place?

◯ Afghanistan
◯ Albania
◯ Algeria
◯ American Samoa
◯ Andorra
◯ Angola
◯ Anguilla
◯ Antarctica
◯ Antigua and Barbuda
◯ Argentina
◯ Armenia
◯ Aruba
◯ Australia
◯ Austria
◯ Azerbaijan
◯ Bahamas
◯ Bahrain
◯ Bangladesh
◯ Barbados
◯ Belarus
◯ Belgium
◯ Belize
◯ Benin
◯ Bermuda
◯ Bhutan
◯ Bolivia
◯ Bosnia and Herzegovina
◯ Botswana
◯ Brazil
◯ British Indian Ocean Territory
◯ British Virgin Islands
◯ Brunei Darussalam
◯ Bulgaria
◯ Burkina Faso
◯ Burundi
◯ Cape Verde
◯ Cambodia
◯ Cameroon
◯ Canada
◯ Cayman Islands
◯ Central African Republic
◯ Chad
◯ Chile
◯ China
◯ Christmas Island
◯ Cocos (Keeling) Islands
◯ Colombia
◯ Comoros
◯ Cook Islands
◯ Costa Rica
◯ Croatia
◯ Cuba
◯ Curaçao
◯ Cyprus
◯ Czech Republic
◯ Democratic Republic of the Congo
◯ Denmark
◯ Djibouti
◯ Dominica
◯ Dominican Republic
◯ East Timor
◯ Ecuador
◯ Egypt
◯ El Salvador
◯ Equatorial Guinea
◯ Eritrea
◯ Estonia
◯ Eswatini (Swaziland)
◯ Ethiopia
◯ Falkland Islands (Malvinas)

◯ Faroe Islands
◯ Fiji
◯ Finland
◯ France
◯ French Polynesia
◯ Gabon
◯ Gambia
◯ Georgia
◯ Germany
◯ Ghana
◯ Gibraltar
◯ Greece
◯ Greenland
◯ Grenada
◯ Guam
◯ Guatemala
◯ Guernsey
◯ Guinea
◯ Guinea-Bissau
◯ Guyana
◯ Haiti
◯ Honduras
◯ Hong Kong
◯ Hungary
◯ Iceland
◯ India
◯ Indonesia
◯ Iran
◯ Iraq
◯ Ireland
◯ Isle of Man
◯ Israel
◯ Italy
◯ Ivory Coast
◯ Jamaica
◯ Japan
◯ Jersey
◯ Jordan
◯ Kazakhstan
◯ Kenya
◯ Kiribati
◯ Kosovo
◯ Kuwait
◯ Kyrgyzstan
◯ Laos
◯ Latvia
◯ Lebanon
◯ Lesotho
◯ Liberia
◯ Libya
◯ Liechtenstein
◯ Lithuania
◯ Luxembourg
◯ Macau
◯ North Macedonia
◯ North Macedonia Madagascar
◯ Malawi
◯ Malaysia
◯ Maldives
◯ Mali
◯ Malta
◯ Marshall Islands
◯ Mauritania
◯ Mauritius
◯ Mayotte
◯ Mexico
◯ Micronesia
◯ Moldova
◯ Monaco
◯ Mongolia
◯ Montenegro

◯ Montserrat
◯ Morocco
◯ Mozambique
◯ Myanmar
◯ Namibia
◯ Nauru
◯ Nepal
◯ Netherlands
◯ Netherlands Antilles
◯ New Caledonia
◯ New Zealand
◯ Nicaragua
◯ Niger
◯ Nigeria
◯ Niue
◯ North Korea
◯ Northern Mariana Islands
◯ Norway
◯ Oman
◯ Pakistan
◯ Palau
◯ Palestine
◯ Panama
◯ Papua New Guinea
◯ Paraguay
◯ Peru
◯ Philippines
◯ Pitcairn
◯ Poland
◯ Portugal
◯ Puerto Rico
◯ Qatar
◯ Republic of the Congo
◯ Réunion
◯ Romania
◯ Russia
◯ Rwanda
◯ Saint Barthélemy
◯ Saint Helena
◯ Saint Kitts and Nevis
◯ Saint Lucia
◯ Saint Martin
◯ Saint Pierre and Miquelon
◯ Saint Vincent and the Grenadines
◯ Samoa
◯ San Marino
◯ São Tomé and Príncipe
◯ audi Arabia
◯ Senegal
◯ Serbia
◯ Seychelles
◯ Sierra Leone
◯ Singapore
◯ Sint Maarten
◯ Slovakia
◯ Slovenia
◯ Solomon Islands
◯ Somalia
◯ South Africa
◯ South Korea
◯ South Sudan
◯ Spain
◯ Sri Lanka
◯ Sudan
◯ Suriname
◯ Svalbard and Jan Mayen
◯ Sweden
◯ Switzerland
◯ Syria
◯ Taiwan
◯ Tajikistan

- ◯ Tanzania
- ◯ Thailand
- ◯ Togo
- ◯ Tokelau
- ◯ Tonga
- ◯ Trinidad and Tobago
- ◯ Tunisia
- ◯ Turkey
- ◯ Turkmenistan
- ◯ Turks and Caicos Islands
- ◯ Tuvalu
- ◯ United States Virgin Islands
- ◯ Uganda
- ◯ Ukraine
- ◯ United Arab Emirates
- ◯ United Kingdom
- ◯ United States of America
- ◯ Uruguay
- ◯ Uzbekistan
- ◯ Vanuatu
- ◯ Venezuela
- ◯ Vietnam
- ◯ Wallis and Futuna
- ◯ Western Sahara
- ◯ Yemen
- ◯ Zambia
- ◯ Zimbabwe
- ◯ Other

9.6.2 How long has it been since the procedure was performed?

- ◯ 0-2 years
- ◯ 3-4 years
- ◯ 5-6 years
- ◯ 7-8 years
- ◯ 9-10 years
- ◯ 11-12 years
- ◯ 13-14 years
- ◯ 15-16 years
- ◯ 17-18 years
- ◯ 19-20 years
- ◯ >20 years

9.6.3 At the time you underwent this in vitro fertilization (IVF), was there any evidence of an endometriotic lesion?

- ◯ Yes
- ◯ No
- ◯ I don't know / I don't remember anymore

9.6.4 Which lesion/s has/have been identified? (check all that apply)

- ☐ Superficial endometriosis
- ☐ Endometrioma ("Chocolate cyst of the ovary")
- ☐ Deep infiltrating endometriosis (i.e. bladder/rectovaginal/ureteral/uterosacral /diaphragmatic nodules)
- ☐ Adenomyosis ("endometriosis of the uterus")
- ☐ I don't remember / I don't know

9.6.5 Where was the procedure performed?

- ◯ Public institution
- ◯ Private institution

9.6.6 Did you have to spend money without being refunded by health insurance?

- ◯ Yes
- ◯ No

9.6.7 How many embryos did you obtain?
- ◯ 0
- ◯ 1
- ◯ 2
- ◯ 3
- ◯ 4
- ◯ 5
- ◯ 6
- ◯ 7
- ◯ 8
- ◯ 9
- ◯ 10
- ◯ 11
- ◯ 12
- ◯ >12
- ◯ I don't know anymore

9.6.8 How many embryo-transfer did you perform?
- ◯ 0
- ◯ 1
- ◯ 2
- ◯ 3
- ◯ 4
- ◯ 5
- ◯ 6
- ◯ 7
- ◯ 8
- ◯ 9
- ◯ 10
- ◯ 11
- ◯ 12
- ◯ >12
- ◯ I don't know anymore

9.6.9 Did you get pregnant?
- ◯ Yes
- ◯ No

9.6.10 Did you deliver at the end of this pregnancy?
- ◯ Yes
- ◯ No

9.6.11 Have you had any complications related to endometriosis during IVF or during pregnancy requiring hospitalization?
- ◯ Yes
- ◯ No

9.6.12 Please check all that apply (Multiple answers possible):
- ☐ Blood in abdomen (hemoperitoneum)
- ☐ Acute bowel obstruction (ileus)
- ☐ Unbearable pain
- ☐ Other complication
- ☐ I don't remember

9.6.13 During and right after this IVF cycle, how would you describe (if any) the change in endometriosis pain?
- ◯ Significantly improved
- ◯ Improved
- ◯ Minimally improved
- ◯ No change
- ◯ Minimally worse
- ◯ Worse
- ◯ Significantly worse

7th cycle

9.7.1In which country did the procedure take place?

- ◯ Afghanistan
- ◯ Albania
- ◯ Algeria
- ◯ American Samoa
- ◯ Andorra
- ◯ Angola
- ◯ Anguilla
- ◯ Antarctica
- ◯ Antigua and Barbuda
- ◯ Argentina
- ◯ Armenia
- ◯ Aruba
- ◯ Australia
- ◯ Austria
- ◯ Azerbaijan
- ◯ Bahamas
- ◯ Bahrain
- ◯ Bangladesh
- ◯ Barbados
- ◯ Belarus
- ◯ Belgium
- ◯ Belize
- ◯ Benin
- ◯ Bermuda
- ◯ Bhutan
- ◯ Bolivia
- ◯ Bosnia and Herzegovina
- ◯ Botswana
- ◯ Brazil
- ◯ British Indian Ocean Territory
- ◯ British Virgin Islands
- ◯ Brunei Darussalam
- ◯ Bulgaria
- ◯ Burkina Faso
- ◯ Burundi
- ◯ Cape Verde
- ◯ Cambodia
- ◯ Cameroon
- ◯ Canada
- ◯ Cayman Islands
- ◯ Central African Republic
- ◯ Chad
- ◯ Chile
- ◯ China
- ◯ Christmas Island
- ◯ Cocos (Keeling) Islands
- ◯ Colombia
- ◯ Comoros
- ◯ Cook Islands
- ◯ Costa Rica
- ◯ Croatia
- ◯ Cuba
- ◯ Curaçao
- ◯ Cyprus
- ◯ Czech Republic
- ◯ Democratic Republic of the Congo
- ◯ Denmark
- ◯ Djibouti
- ◯ Dominica
- ◯ Dominican Republic
- ◯ East Timor
- ◯ Ecuador
- ◯ Egypt
- ◯ El Salvador
- ◯ Equatorial Guinea
- ◯ Eritrea
- ◯ Estonia
- ◯ Eswatini (Swaziland)
- ◯ Ethiopia
- ◯ Falkland Islands (Malvinas)

◯ Faroe Islands
◯ Fiji
◯ Finland
◯ France
◯ French Polynesia
◯ Gabon
◯ Gambia
◯ Georgia
◯ Germany
◯ Ghana
◯ Gibraltar
◯ Greece
◯ Greenland
◯ Grenada
◯ Guam
◯ Guatemala
◯ Guernsey
◯ Guinea
◯ Guinea-Bissau
◯ Guyana
◯ Haiti
◯ Honduras
◯ Hong Kong
◯ Hungary
◯ Iceland
◯ India
◯ Indonesia
◯ Iran
◯ Iraq
◯ Ireland
◯ Isle of Man
◯ Israel
◯ Italy
◯ Ivory Coast
◯ Jamaica
◯ Japan
◯ Jersey
◯ Jordan
◯ Kazakhstan
◯ Kenya
◯ Kiribati
◯ Kosovo
◯ Kuwait
◯ Kyrgyzstan
◯ Laos
◯ Latvia
◯ Lebanon
◯ Lesotho
◯ Liberia
◯ Libya
◯ Liechtenstein
◯ Lithuania
◯ Luxembourg
◯ Macau
◯ North Macedonia
◯ North Macedonia Madagascar
◯ Malawi
◯ Malaysia
◯ Maldives
◯ Mali
◯ Malta
◯ Marshall Islands
◯ Mauritania
◯ Mauritius
◯ Mayotte
◯ Mexico
◯ Micronesia
◯ Moldova
◯ Monaco
◯ Mongolia
◯ Montenegro

◯ Montserrat
◯ Morocco
◯ Mozambique
◯ Myanmar
◯ Namibia
◯ Nauru
◯ Nepal
◯ Netherlands
◯ Netherlands Antilles
◯ New Caledonia
◯ New Zealand
◯ Nicaragua
◯ Niger
◯ Nigeria
◯ Niue
◯ North Korea
◯ Northern Mariana Islands
◯ Norway
◯ Oman
◯ Pakistan
◯ Palau
◯ Palestine
◯ Panama
◯ Papua New Guinea
◯ Paraguay
◯ Peru
◯ Philippines
◯ Pitcairn
◯ Poland
◯ Portugal
◯ Puerto Rico
◯ Qatar
◯ Republic of the Congo
◯ Réunion
◯ Romania
◯ Russia
◯ Rwanda
◯ Saint Barthélemy
◯ Saint Helena
◯ Saint Kitts and Nevis
◯ Saint Lucia
◯ Saint Martin
◯ Saint Pierre and Miquelon
◯ Saint Vincent and the Grenadines
◯ Samoa
◯ San Marino
◯ São Tomé and Príncipe
◯ audi Arabia
◯ Senegal
◯ Serbia
◯ Seychelles
◯ Sierra Leone
◯ Singapore
◯ Sint Maarten
◯ Slovakia
◯ Slovenia
◯ Solomon Islands
◯ Somalia
◯ South Africa
◯ South Korea
◯ South Sudan
◯ Spain
◯ Sri Lanka
◯ Sudan
◯ Suriname
◯ Svalbard and Jan Mayen
◯ Sweden
◯ Switzerland
◯ Syria
◯ Taiwan
◯ Tajikistan

- ◯ Tanzania
- ◯ Thailand
- ◯ Togo
- ◯ Tokelau
- ◯ Tonga
- ◯ Trinidad and Tobago
- ◯ Tunisia
- ◯ Turkey
- ◯ Turkmenistan
- ◯ Turks and Caicos Islands
- ◯ Tuvalu
- ◯ United States Virgin Islands
- ◯ Uganda
- ◯ Ukraine
- ◯ United Arab Emirates
- ◯ United Kingdom
- ◯ United States of America
- ◯ Uruguay
- ◯ Uzbekistan
- ◯ Vanuatu
- ◯ Venezuela
- ◯ Vietnam
- ◯ Wallis and Futuna
- ◯ Western Sahara
- ◯ Yemen
- ◯ Zambia
- ◯ Zimbabwe
- ◯ Other

9.7.2 How long has it been since the procedure was performed?

- ◯ 0-2 years
- ◯ 3-4 years
- ◯ 5-6 years
- ◯ 7-8 years
- ◯ 9-10 years
- ◯ 11-12 years
- ◯ 13-14 years
- ◯ 15-16 years
- ◯ 17-18 years
- ◯ 19-20 years
- ◯ >20 years

9.7.3 At the time you underwent this in vitro fertilization (IVF), was there any evidence of an endometriotic lesion?

- ◯ Yes
- ◯ No
- ◯ I don't know / I don't remember anymore

9.7.4 Which lesion/s has/have been identified? (check all that apply)

- ☐ Superficial endometriosis
- ☐ Endometrioma ("Chocolate cyst of the ovary")
- ☐ Deep infiltrating endometriosis (i.e. bladder/rectovaginal/ureteral/uterosacral /diaphragmatic nodules)
- ☐ Adenomyosis ("endometriosis of the uterus")
- ☐ I don't remember / I don't know

9.7.5 Where was the procedure performed?

- ◯ Public institution
- ◯ Private institution

9.7.6 Did you have to spend money without being refunded by health insurance?

- ◯ Yes
- ◯ No

9.7.7 How many embryos did you obtain?

- ◯ 0
- ◯ 1
- ◯ 2
- ◯ 3
- ◯ 4
- ◯ 5
- ◯ 6
- ◯ 7
- ◯ 8
- ◯ 9
- ◯ 10
- ◯ 11
- ◯ 12
- ◯ >12
- ◯ I don't know anymore

9.7.8 How many embryo-transfer did you perform?

- ◯ 0
- ◯ 1
- ◯ 2
- ◯ 3
- ◯ 4
- ◯ 5
- ◯ 6
- ◯ 7
- ◯ 8
- ◯ 9
- ◯ 10
- ◯ 11
- ◯ 12
- ◯ >12
- ◯ I don't know anymore

9.7.9 Did you get pregnant?

- ◯ Yes
- ◯ No

9.7.10 Did you deliver at the end of this pregnancy?

- ◯ Yes
- ◯ No

9.7.11 Have you had any complications related to endometriosis during IVF or during pregnancy requiring hospitalization?

- ◯ Yes
- ◯ No

9.7.12 Please check all that apply (Multiple answers possible):

- ☐ Blood in abdomen (hemoperitoneum)
- ☐ Acute bowel obstruction (ileus)
- ☐ Unbearable pain
- ☐ Other complication
- ☐ I don't remember

9.7.13 During and right after this IVF cycle, how would you describe (if any) the change in endometriosis pain?

- ◯ Significantly improved
- ◯ Improved
- ◯ Minimally improved
- ◯ No change
- ◯ Minimally worse
- ◯ Worse
- ◯ Significantly worse

8th cycle

9.8.1 In which country did the procedure take place?

◯ Afghanistan
◯ Albania
◯ Algeria
◯ American Samoa
◯ Andorra
◯ Angola
◯ Anguilla
◯ Antarctica
◯ Antigua and Barbuda
◯ Argentina
◯ Armenia
◯ Aruba
◯ Australia
◯ Austria
◯ Azerbaijan
◯ Bahamas
◯ Bahrain
◯ Bangladesh
◯ Barbados
◯ Belarus
◯ Belgium
◯ Belize
◯ Benin
◯ Bermuda
◯ Bhutan
◯ Bolivia
◯ Bosnia and Herzegovina
◯ Botswana
◯ Brazil
◯ British Indian Ocean Territory
◯ British Virgin Islands
◯ Brunei Darussalam
◯ Bulgaria
◯ Burkina Faso
◯ Burundi
◯ Cape Verde
◯ Cambodia
◯ Cameroon
◯ Canada
◯ Cayman Islands
◯ Central African Republic
◯ Chad
◯ Chile
◯ China
◯ Christmas Island
◯ Cocos (Keeling) Islands
◯ Colombia
◯ Comoros
◯ Cook Islands
◯ Costa Rica
◯ Croatia
◯ Cuba
◯ Curaçao
◯ Cyprus
◯ Czech Republic
◯ Democratic Republic of the Congo
◯ Denmark
◯ Djibouti
◯ Dominica
◯ Dominican Republic
◯ East Timor
◯ Ecuador
◯ Egypt
◯ El Salvador
◯ Equatorial Guinea
◯ Eritrea
◯ Estonia
◯ Eswatini (Swaziland)
◯ Ethiopia
◯ Falkland Islands (Malvinas)

◯ Faroe Islands
◯ Fiji
◯ Finland
◯ France
◯ French Polynesia
◯ Gabon
◯ Gambia
◯ Georgia
◯ Germany
◯ Ghana
◯ Gibraltar
◯ Greece
◯ Greenland
◯ Grenada
◯ Guam
◯ Guatemala
◯ Guernsey
◯ Guinea
◯ Guinea-Bissau
◯ Guyana
◯ Haiti
◯ Honduras
◯ Hong Kong
◯ Hungary
◯ Iceland
◯ India
◯ Indonesia
◯ Iran
◯ Iraq
◯ Ireland
◯ Isle of Man
◯ Israel
◯ Italy
◯ Ivory Coast
◯ Jamaica
◯ Japan
◯ Jersey
◯ Jordan
◯ Kazakhstan
◯ Kenya
◯ Kiribati
◯ Kosovo
◯ Kuwait
◯ Kyrgyzstan
◯ Laos
◯ Latvia
◯ Lebanon
◯ Lesotho
◯ Liberia
◯ Libya
◯ Liechtenstein
◯ Lithuania
◯ Luxembourg
◯ Macau
◯ North Macedonia
◯ North Macedonia Madagascar
◯ Malawi
◯ Malaysia
◯ Maldives
◯ Mali
◯ Malta
◯ Marshall Islands
◯ Mauritania
◯ Mauritius
◯ Mayotte
◯ Mexico
◯ Micronesia
◯ Moldova
◯ Monaco
◯ Mongolia
◯ Montenegro

◯ Montserrat
◯ Morocco
◯ Mozambique
◯ Myanmar
◯ Namibia
◯ Nauru
◯ Nepal
◯ Netherlands
◯ Netherlands Antilles
◯ New Caledonia
◯ New Zealand
◯ Nicaragua
◯ Niger
◯ Nigeria
◯ Niue
◯ North Korea
◯ Northern Mariana Islands
◯ Norway
◯ Oman
◯ Pakistan
◯ Palau
◯ Palestine
◯ Panama
◯ Papua New Guinea
◯ Paraguay
◯ Peru
◯ Philippines
◯ Pitcairn
◯ Poland
◯ Portugal
◯ Puerto Rico
◯ Qatar
◯ Republic of the Congo
◯ Réunion
◯ Romania
◯ Russia
◯ Rwanda
◯ Saint Barthélemy
◯ Saint Helena
◯ Saint Kitts and Nevis
◯ Saint Lucia
◯ Saint Martin
◯ Saint Pierre and Miquelon
◯ Saint Vincent and the Grenadines
◯ Samoa
◯ San Marino
◯ São Tomé and Príncipe
◯ audi Arabia
◯ Senegal
◯ Serbia
◯ Seychelles
◯ Sierra Leone
◯ Singapore
◯ Sint Maarten
◯ Slovakia
◯ Slovenia
◯ Solomon Islands
◯ Somalia
◯ South Africa
◯ South Korea
◯ South Sudan
◯ Spain
◯ Sri Lanka
◯ Sudan
◯ Suriname
◯ Svalbard and Jan Mayen
◯ Sweden
◯ Switzerland
◯ Syria
◯ Taiwan
◯ Tajikistan

- ◯ Tanzania
- ◯ Thailand
- ◯ Togo
- ◯ Tokelau
- ◯ Tonga
- ◯ Trinidad and Tobago
- ◯ Tunisia
- ◯ Turkey
- ◯ Turkmenistan
- ◯ Turks and Caicos Islands
- ◯ Tuvalu
- ◯ United States Virgin Islands
- ◯ Uganda
- ◯ Ukraine
- ◯ United Arab Emirates
- ◯ United Kingdom
- ◯ United States of America
- ◯ Uruguay
- ◯ Uzbekistan
- ◯ Vanuatu
- ◯ Venezuela
- ◯ Vietnam
- ◯ Wallis and Futuna
- ◯ Western Sahara
- ◯ Yemen
- ◯ Zambia
- ◯ Zimbabwe
- ◯ Other

9.8.2 How long has it been since the procedure was performed?

- ◯ 0-2 years
- ◯ 3-4 years
- ◯ 5-6 years
- ◯ 7-8 years
- ◯ 9-10 years
- ◯ 11-12 years
- ◯ 13-14 years
- ◯ 15-16 years
- ◯ 17-18 years
- ◯ 19-20 years
- ◯ >20 years

9.8.3 At the time you underwent this in vitro fertilization (IVF), was there any evidence of an endometriotic lesion?

- ◯ Yes
- ◯ No
- ◯ I don't know / I don't remember anymore

9.8.4 Which lesion/s has/have been identified? (check all that apply)

- ☐ Superficial endometriosis
- ☐ Endometrioma ("Chocolate cyst of the ovary")
- ☐ Deep infiltrating endometriosis (i.e. bladder/rectovaginal/ureteral/uterosacral /diaphragmatic nodules)
- ☐ Adenomyosis ("endometriosis of the uterus")
- ☐ I don't remember / I don't know

9.8.5 Where was the procedure performed?

- ◯ Public institution
- ◯ Private institution

9.8.6 Did you have to spend money without being refunded by health insurance?

- ◯ Yes
- ◯ No

9.8.7 How many embryos did you obtain?

- ◯ 0
- ◯ 1
- ◯ 2
- ◯ 3
- ◯ 4
- ◯ 5
- ◯ 6
- ◯ 7
- ◯ 8
- ◯ 9
- ◯ 10
- ◯ 11
- ◯ 12
- ◯ >12
- ◯ I don't know anymore

9.8.8 How many embryo-transfer did you perform?

- ◯ 0
- ◯ 1
- ◯ 2
- ◯ 3
- ◯ 4
- ◯ 5
- ◯ 6
- ◯ 7
- ◯ 8
- ◯ 9
- ◯ 10
- ◯ 11
- ◯ 12
- ◯ >12
- ◯ I don't know anymore

9.8.9 Did you get pregnant?

- ◯ Yes
- ◯ No

9.8.10 Did you deliver at the end of this pregnancy?

- ◯ Yes
- ◯ No

9.8.11 Have you had any complications related to endometriosis during IVF or during pregnancy requiring hospitalization?

- ◯ Yes
- ◯ No

9.8.12 Please check all that apply (Multiple answers possible):

- ☐ Blood in abdomen (hemoperitoneum)
- ☐ Acute bowel obstruction (ileus)
- ☐ Unbearable pain
- ☐ Other complication
- ☐ I don't remember

9.8.13 During and right after this IVF cycle, how would you describe (if any) the change in endometriosis pain?

- ◯ Significantly improved
- ◯ Improved
- ◯ Minimally improved
- ◯ No change
- ◯ Minimally worse
- ◯ Worse
- ◯ Significantly worse

9th cycle

9.9.1 In which country did the procedure take place?

◯ Afghanistan
◯ Albania
◯ Algeria
◯ American Samoa
◯ Andorra
◯ Angola
◯ Anguilla
◯ Antarctica
◯ Antigua and Barbuda
◯ Argentina
◯ Armenia
◯ Aruba
◯ Australia
◯ Austria
◯ Azerbaijan
◯ Bahamas
◯ Bahrain
◯ Bangladesh
◯ Barbados
◯ Belarus
◯ Belgium
◯ Belize
◯ Benin
◯ Bermuda
◯ Bhutan
◯ Bolivia
◯ Bosnia and Herzegovina
◯ Botswana
◯ Brazil
◯ British Indian Ocean Territory
◯ British Virgin Islands
◯ Brunei Darussalam
◯ Bulgaria
◯ Burkina Faso
◯ Burundi
◯ Cape Verde
◯ Cambodia
◯ Cameroon
◯ Canada
◯ Cayman Islands
◯ Central African Republic
◯ Chad
◯ Chile
◯ China
◯ Christmas Island
◯ Cocos (Keeling) Islands
◯ Colombia
◯ Comoros
◯ Cook Islands
◯ Costa Rica
◯ Croatia
◯ Cuba
◯ Curaçao
◯ Cyprus
◯ Czech Republic
◯ Democratic Republic of the Congo
◯ Denmark
◯ Djibouti
◯ Dominica
◯ Dominican Republic
◯ East Timor
◯ Ecuador
◯ Egypt
◯ El Salvador
◯ Equatorial Guinea
◯ Eritrea
◯ Estonia
◯ Eswatini (Swaziland)
◯ Ethiopia
◯ Falkland Islands (Malvinas)

◯ Faroe Islands
◯ Fiji
◯ Finland
◯ France
◯ French Polynesia
◯ Gabon
◯ Gambia
◯ Georgia
◯ Germany
◯ Ghana
◯ Gibraltar
◯ Greece
◯ Greenland
◯ Grenada
◯ Guam
◯ Guatemala
◯ Guernsey
◯ Guinea
◯ Guinea-Bissau
◯ Guyana
◯ Haiti
◯ Honduras
◯ Hong Kong
◯ Hungary
◯ Iceland
◯ India
◯ Indonesia
◯ Iran
◯ Iraq
◯ Ireland
◯ Isle of Man
◯ Israel
◯ Italy
◯ Ivory Coast
◯ Jamaica
◯ Japan
◯ Jersey
◯ Jordan
◯ Kazakhstan
◯ Kenya
◯ Kiribati
◯ Kosovo
◯ Kuwait
◯ Kyrgyzstan
◯ Laos
◯ Latvia
◯ Lebanon
◯ Lesotho
◯ Liberia
◯ Libya
◯ Liechtenstein
◯ Lithuania
◯ Luxembourg
◯ Macau
◯ North Macedonia
◯ North Macedonia Madagascar
◯ Malawi
◯ Malaysia
◯ Maldives
◯ Mali
◯ Malta
◯ Marshall Islands
◯ Mauritania
◯ Mauritius
◯ Mayotte
◯ Mexico
◯ Micronesia
◯ Moldova
◯ Monaco
◯ Mongolia
◯ Montenegro

◯ Montserrat
◯ Morocco
◯ Mozambique
◯ Myanmar
◯ Namibia
◯ Nauru
◯ Nepal
◯ Netherlands
◯ Netherlands Antilles
◯ New Caledonia
◯ New Zealand
◯ Nicaragua
◯ Niger
◯ Nigeria
◯ Niue
◯ North Korea
◯ Northern Mariana Islands
◯ Norway
◯ Oman
◯ Pakistan
◯ Palau
◯ Palestine
◯ Panama
◯ Papua New Guinea
◯ Paraguay
◯ Peru
◯ Philippines
◯ Pitcairn
◯ Poland
◯ Portugal
◯ Puerto Rico
◯ Qatar
◯ Republic of the Congo
◯ Réunion
◯ Romania
◯ Russia
◯ Rwanda
◯ Saint Barthélemy
◯ Saint Helena
◯ Saint Kitts and Nevis
◯ Saint Lucia
◯ Saint Martin
◯ Saint Pierre and Miquelon
◯ Saint Vincent and the Grenadines
◯ Samoa
◯ San Marino
◯ São Tomé and Príncipe
◯ audi Arabia
◯ Senegal
◯ Serbia
◯ Seychelles
◯ Sierra Leone
◯ Singapore
◯ Sint Maarten
◯ Slovakia
◯ Slovenia
◯ Solomon Islands
◯ Somalia
◯ South Africa
◯ South Korea
◯ South Sudan
◯ Spain
◯ Sri Lanka
◯ Sudan
◯ Suriname
◯ Svalbard and Jan Mayen
◯ Sweden
◯ Switzerland
◯ Syria
◯ Taiwan
◯ Tajikistan

- ◯ Tanzania
- ◯ Thailand
- ◯ Togo
- ◯ Tokelau
- ◯ Tonga
- ◯ Trinidad and Tobago
- ◯ Tunisia
- ◯ Turkey
- ◯ Turkmenistan
- ◯ Turks and Caicos Islands
- ◯ Tuvalu
- ◯ United States Virgin Islands
- ◯ Uganda
- ◯ Ukraine
- ◯ United Arab Emirates
- ◯ United Kingdom
- ◯ United States of America
- ◯ Uruguay
- ◯ Uzbekistan
- ◯ Vanuatu
- ◯ Venezuela
- ◯ Vietnam
- ◯ Wallis and Futuna
- ◯ Western Sahara
- ◯ Yemen
- ◯ Zambia
- ◯ Zimbabwe
- ◯ Other

9.9.2 How long has it been since the procedure was performed?

- ◯ 0-2 years
- ◯ 3-4 years
- ◯ 5-6 years
- ◯ 7-8 years
- ◯ 9-10 years
- ◯ 11-12 years
- ◯ 13-14 years
- ◯ 15-16 years
- ◯ 17-18 years
- ◯ 19-20 years
- ◯ >20 years

9.9.3 At the time you underwent this in vitro fertilization (IVF), was there any evidence of an endometriotic lesion?

- ◯ Yes
- ◯ No
- ◯ I don't know / I don't remember anymore

9.9.4 Which lesion/s has/have been identified? (check all that apply)

- ☐ Superficial Endometrioma
- ☐ Endometrioma ("Chocolate ovarian cyst")
- ☐ Deep infiltrating endometriosis (i.e. bladder/rectovaginal/ureteral/uterosacral /diaphragmatic nodules)
- ☐ Adenomyosis ("endometriosis of the uterus")
- ☐ I don't remember / I don't know

9.3 Where was the procedure performed?

- ◯ Public institution
- ◯ Private institution

9.9.5 Did you have to spend money without being refunded by health insurance?

- ◯ Yes
- ◯ No

9.9.6 How many embryos did you obtain?

- ◯ 0
- ◯ 1
- ◯ 2
- ◯ 3
- ◯ 4
- ◯ 5
- ◯ 6
- ◯ 7
- ◯ 8
- ◯ 9
- ◯ 10
- ◯ 11
- ◯ 12
- ◯ >12
- ◯ I don't know anymore

9.9.7 How many embryo-transfer did you perform?

- ◯ 0
- ◯ 1
- ◯ 2
- ◯ 3
- ◯ 4
- ◯ 5
- ◯ 6
- ◯ 7
- ◯ 8
- ◯ 9
- ◯ 10
- ◯ 11
- ◯ 12
- ◯ >12
- ◯ I don't know anymore

9.9.8 Did you get pregnant?

- ◯ Yes
- ◯ No

9.9.9 Did you deliver at the end of this pregnancy?

- ◯ Yes
- ◯ No

9.9.11 Have you had any complications related to endometriosis during IVF or during pregnancy requiring hospitalization?

- ◯ Yes
- ◯ No

9.9.12 Please check all that apply (Multiple answers possible):

- ☐ Blood in abdomen (hemoperitoneum)
- ☐ Acute bowel obstruction (ileus)
- ☐ Unbearable pain
- ☐ Other complication
- ☐ I don't remember

9.9.13 During and right after this IVF cycle, how would you describe (if any) the change in endometriosis pain?

- ◯ Significantly improved
- ◯ Improved
- ◯ Minimally improved
- ◯ No change
- ◯ Minimally worse
- ◯ Worse
- ◯ Significantly worse

10th cycle

9.10.1 In which country did the procedure take place?

◯ Afghanistan
◯ Albania
◯ Algeria
◯ American Samoa
◯ Andorra
◯ Angola
◯ Anguilla
◯ Antarctica
◯ Antigua and Barbuda
◯ Argentina
◯ Armenia
◯ Aruba
◯ Australia
◯ Austria
◯ Azerbaijan
◯ Bahamas
◯ Bahrain
◯ Bangladesh
◯ Barbados
◯ Belarus
◯ Belgium
◯ Belize
◯ Benin
◯ Bermuda
◯ Bhutan
◯ Bolivia
◯ Bosnia and Herzegovina
◯ Botswana
◯ Brazil
◯ British Indian Ocean Territory
◯ British Virgin Islands
◯ Brunei Darussalam
◯ Bulgaria
◯ Burkina Faso
◯ Burundi
◯ Cape Verde
◯ Cambodia
◯ Cameroon
◯ Canada
◯ Cayman Islands
◯ Central African Republic
◯ Chad
◯ Chile
◯ China
◯ Christmas Island
◯ Cocos (Keeling) Islands
◯ Colombia
◯ Comoros
◯ Cook Islands
◯ Costa Rica
◯ Croatia
◯ Cuba
◯ Curaçao
◯ Cyprus
◯ Czech Republic
◯ Democratic Republic of the Congo
◯ Denmark
◯ Djibouti
◯ Dominica
◯ Dominican Republic
◯ East Timor
◯ Ecuador
◯ Egypt
◯ El Salvador
◯ Equatorial Guinea
◯ Eritrea
◯ Estonia
◯ Eswatini (Swaziland)
◯ Ethiopia
◯ Falkland Islands (Malvinas)

◯ Faroe Islands
◯ Fiji
◯ Finland
◯ France
◯ French Polynesia
◯ Gabon
◯ Gambia
◯ Georgia
◯ Germany
◯ Ghana
◯ Gibraltar
◯ Greece
◯ Greenland
◯ Grenada
◯ Guam
◯ Guatemala
◯ Guernsey
◯ Guinea
◯ Guinea-Bissau
◯ Guyana
◯ Haiti
◯ Honduras
◯ Hong Kong
◯ Hungary
◯ Iceland
◯ India
◯ Indonesia
◯ Iran
◯ Iraq
◯ Ireland
◯ Isle of Man
◯ Israel
◯ Italy
◯ Ivory Coast
◯ Jamaica
◯ Japan
◯ Jersey
◯ Jordan
◯ Kazakhstan
◯ Kenya
◯ Kiribati
◯ Kosovo
◯ Kuwait
◯ Kyrgyzstan
◯ Laos
◯ Latvia
◯ Lebanon
◯ Lesotho
◯ Liberia
◯ Libya
◯ Liechtenstein
◯ Lithuania
◯ Luxembourg
◯ Macau
◯ North Macedonia
◯ North Macedonia Madagascar
◯ Malawi
◯ Malaysia
◯ Maldives
◯ Mali
◯ Malta
◯ Marshall Islands
◯ Mauritania
◯ Mauritius
◯ Mayotte
◯ Mexico
◯ Micronesia
◯ Moldova
◯ Monaco
◯ Mongolia
◯ Montenegro

◯ Montserrat
◯ Morocco
◯ Mozambique
◯ Myanmar
◯ Namibia
◯ Nauru
◯ Nepal
◯ Netherlands
◯ Netherlands Antilles
◯ New Caledonia
◯ New Zealand
◯ Nicaragua
◯ Niger
◯ Nigeria
◯ Niue
◯ North Korea
◯ Northern Mariana Islands
◯ Norway
◯ Oman
◯ Pakistan
◯ Palau
◯ Palestine
◯ Panama
◯ Papua New Guinea
◯ Paraguay
◯ Peru
◯ Philippines
◯ Pitcairn
◯ Poland
◯ Portugal
◯ Puerto Rico
◯ Qatar
◯ Republic of the Congo
◯ Réunion
◯ Romania
◯ Russia
◯ Rwanda
◯ Saint Barthélemy
◯ Saint Helena
◯ Saint Kitts and Nevis
◯ Saint Lucia
◯ Saint Martin
◯ Saint Pierre and Miquelon
◯ Saint Vincent and the Grenadines
◯ Samoa
◯ San Marino
◯ São Tomé and Príncipe
◯ audi Arabia
◯ Senegal
◯ Serbia
◯ Seychelles
◯ Sierra Leone
◯ Singapore
◯ Sint Maarten
◯ Slovakia
◯ Slovenia
◯ Solomon Islands
◯ Somalia
◯ South Africa
◯ South Korea
◯ South Sudan
◯ Spain
◯ Sri Lanka
◯ Sudan
◯ Suriname
◯ Svalbard and Jan Mayen
◯ Sweden
◯ Switzerland
◯ Syria
◯ Taiwan
◯ Tajikistan

◯ Tanzania
◯ Thailand
◯ Togo
◯ Tokelau
◯ Tonga
◯ Trinidad and Tobago
◯ Tunisia
◯ Turkey
◯ Turkmenistan
◯ Turks and Caicos Islands
◯ Tuvalu
◯ United States Virgin Islands
◯ Uganda
◯ Ukraine
◯ United Arab Emirates
◯ United Kingdom
◯ United States of America
◯ Uruguay
◯ Uzbekistan
◯ Vanuatu
◯ Venezuela
◯ Vietnam
◯ Wallis and Futuna
◯ Western Sahara
◯ Yemen
◯ Zambia
◯ Zimbabwe
◯ Other

9.10.2 How long has it been since the procedure was performed?

◯ 0-2 years
◯ 3-4 years
◯ 5-6 years
◯ 7-8 years
◯ 9-10 years
◯ 11-12 years
◯ 13-14 years
◯ 15-16 years
◯ 17-18 years
◯ 19-20 years
◯ >20 years

9.10.3 At the time you underwent this in vitro fertilization (IVF), was there any evidence of an endometriotic lesion?

◯ Yes
◯ No
◯ I don't know / I don't remember anymore

9.10.4 Which lesion/s has/have been identified? (check all that apply)

☐ Superficial endometriosis
☐ Endometrioma ("Chocolate cyst of the ovary")
☐ Deep infiltrating endometriosis (i.e. bladder/rectovaginal/ureteral/uterosacral /diaphragmatic nodules)
☐ Adenomyosis ("endometriosis of the uterus")
☐ I don't remember / I don't know

9.10.5 Where was the procedure performed?

◯ Public institution
◯ Private institution

9.10.6 Did you have to spend money without being refunded by health insurance?

◯ Yes
◯ No

9.10.7 How many embryos did you obtain?
- ◯ 0
- ◯ 1
- ◯ 2
- ◯ 3
- ◯ 4
- ◯ 5
- ◯ 6
- ◯ 7
- ◯ 8
- ◯ 9
- ◯ 10
- ◯ 11
- ◯ 12
- ◯ >12
- ◯ I don't know anymore

9.10.8 How many embryo-transfer did you perform?
- ◯ 0
- ◯ 1
- ◯ 2
- ◯ 3
- ◯ 4
- ◯ 5
- ◯ 6
- ◯ 7
- ◯ 8
- ◯ 9
- ◯ 10
- ◯ 11
- ◯ 12
- ◯ >12
- ◯ I don't know anymore

9.10.9 Did you get pregnant?
- ◯ Yes
- ◯ No

9.10.10 Did you deliver at the end of this pregnancy?
- ◯ Yes
- ◯ No

9.10.11 Have you had any complications related to endometriosis during IVF or during pregnancy requiring hospitalization?
- ◯ Yes
- ◯ No

9.10.12 Please check all that apply (Multiple answers possible):
- ☐ Blood in abdomen (hemoperitoneum)
- ☐ Acute bowel obstruction (ileus)
- ☐ Unbearable pain
- ☐ Other complication
- ☐ I don't remember

9.10.13 During and right after this IVF cycle, how would you describe (if any) the change in endometriosis pain?
- ◯ Significantly improved
- ◯ Improved
- ◯ Minimally improved
- ◯ No change
- ◯ Minimally worse
- ◯ Worse
- ◯ Significantly worse

11th cycle

9.11.1 In which country did the procedure take place?

◯ Afghanistan
◯ Albania
◯ Algeria
◯ American Samoa
◯ Andorra
◯ Angola
◯ Anguilla
◯ Antarctica
◯ Antigua and Barbuda
◯ Argentina
◯ Armenia
◯ Aruba
◯ Australia
◯ Austria
◯ Azerbaijan
◯ Bahamas
◯ Bahrain
◯ Bangladesh
◯ Barbados
◯ Belarus
◯ Belgium
◯ Belize
◯ Benin
◯ Bermuda
◯ Bhutan
◯ Bolivia
◯ Bosnia and Herzegovina
◯ Botswana
◯ Brazil
◯ British Indian Ocean Territory
◯ British Virgin Islands
◯ Brunei Darussalam
◯ Bulgaria
◯ Burkina Faso
◯ Burundi
◯ Cape Verde
◯ Cambodia
◯ Cameroon
◯ Canada
◯ Cayman Islands
◯ Central African Republic
◯ Chad
◯ Chile
◯ China
◯ Christmas Island
◯ Cocos (Keeling) Islands
◯ Colombia
◯ Comoros
◯ Cook Islands
◯ Costa Rica
◯ Croatia
◯ Cuba
◯ Curaçao
◯ Cyprus
◯ Czech Republic
◯ Democratic Republic of the Congo
◯ Denmark
◯ Djibouti
◯ Dominica
◯ Dominican Republic
◯ East Timor
◯ Ecuador
◯ Egypt
◯ El Salvador
◯ Equatorial Guinea
◯ Eritrea
◯ Estonia
◯ Eswatini (Swaziland)
◯ Ethiopia
◯ Falkland Islands (Malvinas)

◯ Faroe Islands
◯ Fiji
◯ Finland
◯ France
◯ French Polynesia
◯ Gabon
◯ Gambia
◯ Georgia
◯ Germany
◯ Ghana
◯ Gibraltar
◯ Greece
◯ Greenland
◯ Grenada
◯ Guam
◯ Guatemala
◯ Guernsey
◯ Guinea
◯ Guinea-Bissau
◯ Guyana
◯ Haiti
◯ Honduras
◯ Hong Kong
◯ Hungary
◯ Iceland
◯ India
◯ Indonesia
◯ Iran
◯ Iraq
◯ Ireland
◯ Isle of Man
◯ Israel
◯ Italy
◯ Ivory Coast
◯ Jamaica
◯ Japan
◯ Jersey
◯ Jordan
◯ Kazakhstan
◯ Kenya
◯ Kiribati
◯ Kosovo
◯ Kuwait
◯ Kyrgyzstan
◯ Laos
◯ Latvia
◯ Lebanon
◯ Lesotho
◯ Liberia
◯ Libya
◯ Liechtenstein
◯ Lithuania
◯ Luxembourg
◯ Macau
◯ North Macedonia
◯ North Macedonia Madagascar
◯ Malawi
◯ Malaysia
◯ Maldives
◯ Mali
◯ Malta
◯ Marshall Islands
◯ Mauritania
◯ Mauritius
◯ Mayotte
◯ Mexico
◯ Micronesia
◯ Moldova
◯ Monaco
◯ Mongolia
◯ Montenegro

◯ Montserrat
◯ Morocco
◯ Mozambique
◯ Myanmar
◯ Namibia
◯ Nauru
◯ Nepal
◯ Netherlands
◯ Netherlands Antilles
◯ New Caledonia
◯ New Zealand
◯ Nicaragua
◯ Niger
◯ Nigeria
◯ Niue
◯ North Korea
◯ Northern Mariana Islands
◯ Norway
◯ Oman
◯ Pakistan
◯ Palau
◯ Palestine
◯ Panama
◯ Papua New Guinea
◯ Paraguay
◯ Peru
◯ Philippines
◯ Pitcairn
◯ Poland
◯ Portugal
◯ Puerto Rico
◯ Qatar
◯ Republic of the Congo
◯ Réunion
◯ Romania
◯ Russia
◯ Rwanda
◯ Saint Barthélemy
◯ Saint Helena
◯ Saint Kitts and Nevis
◯ Saint Lucia
◯ Saint Martin
◯ Saint Pierre and Miquelon
◯ Saint Vincent and the Grenadines
◯ Samoa
◯ San Marino
◯ São Tomé and Príncipe
◯ audi Arabia
◯ Senegal
◯ Serbia
◯ Seychelles
◯ Sierra Leone
◯ Singapore
◯ Sint Maarten
◯ Slovakia
◯ Slovenia
◯ Solomon Islands
◯ Somalia
◯ South Africa
◯ South Korea
◯ South Sudan
◯ Spain
◯ Sri Lanka
◯ Sudan
◯ Suriname
◯ Svalbard and Jan Mayen
◯ Sweden
◯ Switzerland
◯ Syria
◯ Taiwan
◯ Tajikistan

- ◯ Tanzania
- ◯ Thailand
- ◯ Togo
- ◯ Tokelau
- ◯ Tonga
- ◯ Trinidad and Tobago
- ◯ Tunisia
- ◯ Turkey
- ◯ Turkmenistan
- ◯ Turks and Caicos Islands
- ◯ Tuvalu
- ◯ United States Virgin Islands
- ◯ Uganda
- ◯ Ukraine
- ◯ United Arab Emirates
- ◯ United Kingdom
- ◯ United States of America
- ◯ Uruguay
- ◯ Uzbekistan
- ◯ Vanuatu
- ◯ Venezuela
- ◯ Vietnam
- ◯ Wallis and Futuna
- ◯ Western Sahara
- ◯ Yemen
- ◯ Zambia
- ◯ Zimbabwe
- ◯ Other

9.11.2 How long has it been since the procedure was performed?

- ◯ 0-2 years
- ◯ 3-4 years
- ◯ 5-6 years
- ◯ 7-8 years
- ◯ 9-10 years
- ◯ 11-12 years
- ◯ 13-14 years
- ◯ 15-16 years
- ◯ 17-18 years
- ◯ 19-20 years
- ◯ >20 years

9.11.3 At the time you underwent this in vitro fertilization (IVF), was there any evidence of an endometriotic lesion?

- ◯ Yes
- ◯ No
- ◯ I don't know / I don't remember anymore

9.11.4 Which lesion/s has/have been identified? (check all that apply)

- ☐ Superficial endometriosis
- ☐ Endometrioma ("Chocolate cyst of the ovary")
- ☐ Deep infiltrating endometriosis (i.e. bladder/rectovaginal/ureteral/uterosacral /diaphragmatic nodules)
- ☐ Adenomyosis ("endometriosis of the uterus")
- ☐ I don't remember / I don't know

9.11.5 Where was the procedure performed?

- ◯ Public institution
- ◯ Private institution

9.11.6 Did you have to spend money without being refunded by health insurance?

- ◯ Yes
- ◯ No

9.11.7 How many embryos did you obtain?

- ◯ 0
- ◯ 1
- ◯ 2
- ◯ 3
- ◯ 4
- ◯ 5
- ◯ 6
- ◯ 7
- ◯ 8
- ◯ 9
- ◯ 10
- ◯ 11
- ◯ 12
- ◯ >12
- ◯ I don't know anymore

9.11.8 How many embryo-transfer did you perform?

- ◯ 0
- ◯ 1
- ◯ 2
- ◯ 3
- ◯ 4
- ◯ 5
- ◯ 6
- ◯ 7
- ◯ 8
- ◯ 9
- ◯ 10
- ◯ 11
- ◯ 12
- ◯ >12
- ◯ I don't know anymore

9.11.9 Did you get pregnant?

- ◯ Yes
- ◯ No

9.11.10 Did you deliver at the end of this pregnancy?

- ◯ Yes
- ◯ No

9.11.11 Have you had any complications related to endometriosis during IVF or during pregnancy requiring hospitalization?

- ◯ Yes
- ◯ No

9.11.12 Please check all that apply (Multiple answers possible):

- ☐ Blood in abdomen (hemoperitoneum)
- ☐ Acute bowel obstruction (ileus)
- ☐ Unbearable pain
- ☐ Other complication
- ☐ I don't remember

9.11.13 During and right after this IVF cycle, how would you describe (if any) the change in endometriosis pain?

- ◯ Significantly improved
- ◯ Improved
- ◯ Minimally improved
- ◯ No change
- ◯ Minimally worse
- ◯ Worse
- ◯ Significantly worse

Please answer the following questions concerning your health and IVF in general.

10 Which phase of IVF treatment do you recall as the worst experience in term of pain?

- ◯ Ovarian stimulation (include ultrasound monitoring)
- ◯ Trigger injection
- ◯ Oocyte retrieval
- ◯ Embryo-transfer
- ◯ First phase of pregnancy
- ◯ The first menstruation after the oocyte retrieval
- ◯ I don't know

---

11 Are you currently under hormonal therapy for the treatment of endometriosis pain?

- ◯ Yes
- ◯ No

---

12 Are you currently taking analgesic (painkiller) treatments to manage the pain of endometriosis?

- ◯ Yes
- ◯ No

12.1 Please check all that apply:

- ☐ Paracetamol (Acetaminophen)
- ☐ Non-steroidal anti-inflammatory drugs (NSAIDs) (Ibuprofen, Diclofenac, Naproxen, Celecoxib)
- ☐ Mild opioid analgesics (Tramadol, Codeine)
- ☐ Strong opioid analgesics (Morphine, Oxycodone)
- ☐ Other

---

13 Are you actually practicing alternative medicine to manage endometriosis pain?

- ◯ Yes
- ◯ No

13.1 Please check all that apply:

- ☐ Acupuncture
- ☐ Hypnosis
- ☐ Osteopathy/Physiotherapy
- ☐ Herbal remedies
- ☐ Psychotherapy
- ☐ Relaxing therapy
- ☐ Chiropractic
- ☐ Transcutaneous electric nerve stimulation (TENS)
- ☐ Others

---

14 Have you ever been diagnosed with any of the following diseases? (check all that apply)

- ☐ Fibromyalgia
- ☐ Interstitial cystitis (bladder pain syndrome)
- ☐ Irritable bowel syndrome
- ☐ Migraine
- ☐ Chronic fatigue syndrome
- ☐ Inflammatory bowel disease
- ☐ Chronic low back pain
- ☐ I have never been diagnosed with any of these illness

---

15 Following IVF, did you resort to surgery to treat endometriosis?

- ◯ Yes
- ◯ No

15.1 How many years after IVF did you undergo surgery?

- ◯ 0-2 years
- ◯ 3-5 years
- ◯ 6-8 years
- ◯ 9-10 years
- ◯ >10 years
- ◯ I don't remember

15.2 Which therapeutic procedures have been performed? (check all that apply)

- ☐ Superficial peritoneal endometriosis excision
- ☐ Cystectomy (removal of the cyst)
- ☐ Ovariectomy (removal of the ovary)
- ☐ Nodules excision (i.e.

er/rectovaginal/ureteral/uterosacral/diaphragmatic nodules)
- ☐ Bowel resection
- ☐ Hysterectomy (removal of the uterus)
- ☐ I don't know / I don't remember anymore

16 How could you rate right now your dyspareunia (pain during sexual intercourse)?

0 (no pain) — 10 (worst imaginable pain)

(Place a mark on the scale above)

17 Since IVF treatment/s how would you describe the change (if any) in term of dyspareunia?

- ◯ Significantly Improved
- ◯ Improved
- ◯ Minimally improved
- ◯ No change
- ◯ Minimally worse
- ◯ Worse
- ◯ Significantly worse

18 How could you rate right now your pelvic pain (average level in the last 30 days) ?

0 (no pain) — 10 (worst imaginable pain)

(Place a mark on the scale above)

19 Since IVF treatment/s how would you describe the change (if any) in term of pelvic pain?

- ◯ Significantly improved
- ◯ Improved
- ◯ Minimally improved
- ◯ No change
- ◯ Minimally worse
- ◯ Worse
- ◯ Significantly worse

20 Do you currently have your periods?

- ◯ Yes
- ◯ No

20.1 How could you rate your dysmenorrhea (pain during menstruation)?

0 (no pain) — 10 (worst imaginable pain)

(Place a mark on the scale above)

20.2 Since IVF treatment/s how would you describe the change (if any) in term of dysmenorrhea?

- ◯ Improved
- ◯ much improved
- ◯ minimally improved
- ◯ no change
- ◯ minimally worse
- ◯ much worse
- ◯ very much worse

21 Did infertility treatment negatively affect your mood?

- ◯ Always
- ◯ Very Often
- ◯ Quite often
- ◯ Seldom
- ◯ Never

22 Have you ever been diagnosed with any of the following psychiatric disorders? (check all that apply)

- ☐ Depression
- ☐ Anxiety disorders
- ☐ Eating disorders
- ☐ Addictive behaviours (opioids addiction or drugs)
- ☐ Other
- ☐ I have never been diagnosed with any of these disorders

23 Were you satisfied with the quality of medical services available to address your physical and emotional need during in vitro fertilization?

- ◯ Never
- ◯ Seldom
- ◯ Quite often
- ◯ Very Often
- ◯ Always

24 Have IVF had a negative impact on your relationship with your partner?

- ◯ Immensely
- ◯ A lot
- ◯ Moderately
- ◯ Slightly
- ◯ Not at all

25 Do you still wish to have children in the future?

- ◯ Yes
- ◯ No
