# Supplementary material for: IVF in endometriosis: emerging evidence of exacerbation of pelvic pain and potential predictors
Source: Hum Reprod Open. 2026 Mar 27;2026(2):hoag027. doi: 10.1093/hropen/hoag027 (PMC13091650; doi:10.1093/hropen/hoag027)
Supplement: hoag027_Supplementary_Data [file hoag027_supplementary_data.zip › HROPEN-25-0319.R2 - Supplementary Tables FInal.docx]

Supplementary Table S1. Univariate logistic regression for associations with pelvic pain worsening after IVF.

| **Variable** | **N** | **OR (95% CI)** | **p-value** | **Sig.** |
| --- | --- | --- | --- | --- |
|  |  |  |  |  |
|  |  |  |  |  |
| **Demographics & Socioeconomics** |  | | | |
| Age at survey (years) | 530 | 1.01 (0.98–1.03) | 0.632 |  |
| Out-of-pocket IVF costs | 527 | 1.10 (0.75–1.62) | 0.636 |  |
| **Geographic region (ref: Europe)** | 530 |  |  |  |
| North America | 530 | 1.46 (0.90–2.39) | 0.123 |  |
| Other | 530 | 2.51 (1.23–5.45) | **0.014** | ***** |
| **Education level (ref: Primary school)** | 528 |  |  |  |
| Secondary education | 528 | 1.30 (0.46–3.73) | 0.618 |  |
| Tertiary education | 528 | 1.12 (0.42–3.05) | 0.812 |  |
| **Reproductive history** |  | | | |
| Birth before IVF | 530 | 2.30 (1.02–5.70) | 0.055 |  |
| Age at first IVF cycle (years) | 527 | 1.04 (1.00–1.08) | 0.057 |  |
| Time since first IVF cycle (years) | 527 | 0.98 (0.94–1.02) | 0.279 |  |
| Number of IVF cycles | 530 | 0.97 (0.87–1.09) | 0.613 |  |
| **Endometriosis & surgery** |  | | | |
| **rASRM stage (includes 'no surgery') (ref: No surgery)** | 530 |  |  |  |
| rASRM I | 530 | 0.95 (0.25–3.54) | 0.936 |  |
| rASRM II | 530 | 0.81 (0.35–1.87) | 0.627 |  |
| rASRM III | 530 | 1.06 (0.57–1.97) | 0.858 |  |
| rASRM IV | 530 | 1.10 (0.72–1.68) | 0.657 |  |
| Unknown surgery | 530 | 0.65 (0.37–1.10) | 0.111 |  |
| Superficial endometriosis | 378 | 0.89 (0.58–1.36) | 0.583 |  |
| Ovarian endometrioma | 378 | 0.83 (0.55–1.24) | 0.354 |  |
| Deep endometriosis | 378 | 1.76 (1.12–2.81) | **0.016** | ***** |
| Adenomyosis | 378 | 1.02 (0.68–1.53) | 0.918 |  |
| Surgery before IVF | 530 | 0.95 (0.65–1.38) | 0.784 |  |
| **IVF cycle events & experience** |  | | | |
| Any complication during IVF cycle | 530 | 1.25 (0.79–1.99) | 0.334 |  |
| Hemoperitoneum | 530 | 0.43 (0.12–1.34) | 0.164 |  |
| Ileus | 530 | 1.48 (0.24–11.33) | 0.667 |  |
| Other complication | 530 | 1.37 (0.75–2.53) | 0.311 |  |
| **Most painful step of IVF (ref: Ovarian stimulation (include ultrasound))** | 530 |  |  |  |
| Trigger injection | 530 | 1.03 (0.46–2.33) | 0.936 |  |
| Oocyte retrieval | 530 | 0.63 (0.41–0.97) | **0.038** | ***** |
| Embryo-transfer | 530 | 1.10 (0.50–2.46) | 0.817 |  |
| First phase of pregnancy | 530 | 0.84 (0.31–2.26) | 0.726 |  |
| I don't know | 530 | 0.50 (0.24–1.02) | 0.061 |  |
| 1st menstruation after the oocyte retrieval | 530 | 1.40 (0.81–2.45) | 0.236 |  |
| Pelvic pain worsening soon after IVF | 528 | 6.16 (4.24–9.04) | **<0.001** | ******* |
| **Pain measures (current VAS)** |  | | | |
| Current pelvic pain (0–10) | 489 | 1.45 (1.34–1.58) | **<0.001** | ******* |
| Current dyspareunia (0–10) | 492 | 1.15 (1.07–1.23) | **<0.001** | ******* |
| Current menstrual pain (0–10) | 215 | 1.20 (1.08–1.34) | **0.001** | ****** |
| Currently menstruating | 520 | 1.69 (1.19–2.39) | **0.003** | ****** |
| **Reproductive outcomes (totals)** |  | | | |
| Total embryos obtained | 530 | 0.96 (0.93–0.99) | **0.018** | ***** |
| Total embryo transfers | 530 | 0.95 (0.89–1.00) | 0.124 |  |
| Total pregnancies | 530 | 0.55 (0.43–0.70) | **<0.001** | ******* |
| Live births following IVF (total) | 530 | 0.45 (0.33–0.62) | **<0.001** | ******* |
| Number of miscarriages | 530 | 0.84 (0.63–1.12) | 0.238 |  |
| Future pregnancy wish | 372 | 0.78 (0.10–4.77) | 0.788 |  |
| **Comorbidities & psychiatric** |  | | | |
| Psychiatric disorder (any) | 525 | 1.22 (0.86–1.73) | 0.258 |  |
| Depression | 530 | 1.30 (0.89–1.92) | 0.176 |  |
| Anxiety disorder | 530 | 1.50 (1.03–2.19) | **0.037** | ***** |
| Bladder pain syndrome | 530 | 3.45 (1.53–8.82) | **0.005** | ****** |
| Irritable bowel syndrome | 530 | 1.65 (1.10–2.51) | **0.017** | ***** |
| Migraine | 530 | 0.82 (0.55–1.21) | 0.317 |  |
| Fibromyalgia | 530 | 2.18 (0.90–5.78) | 0.095 |  |
| Chronic fatigue syndrome | 530 | 1.30 (0.75–2.27) | 0.358 |  |
| Chronic inflammatory bowel disease | 530 | 1.33 (0.71–2.50) | 0.373 |  |
| Chronic back pain | 530 | 1.14 (0.74–1.77) | 0.556 |  |
| **Treatments & analgesics (current)** |  | | | |
| Current hormonal treatment | 530 | 1.04 (0.73–1.48) | 0.823 |  |
| Current analgesic use (any) | 530 | 3.12 (2.19–4.46) | **<0.001** | ******* |
| Paracetamol | 530 | 1.66 (1.12–2.49) | **0.012** | ***** |
| NSAIDs | 530 | 2.74 (1.92–3.92) | **<0.001** | ******* |
| Strong opioids | 530 | 7.22 (1.99–46.29) | **0.009** | ****** |
| Light opioids | 530 | 2.15 (1.25–3.81) | **0.007** | ****** |
| Other analgesic | 530 | 1.48 (0.76–2.98) | 0.254 |  |
| **Care experience & psychosocial** |  | | | |
| **Satisfaction with services (ref: Never)** | 530 |  |  |  |
| Seldom | 530 | 0.55 (0.30–0.98) | **0.045** | ***** |
| Quite often | 530 | 0.42 (0.23–0.76) | **0.005** | ****** |
| Very often | 530 | 0.29 (0.15–0.54) | **<0.001** | ******* |
| Always | 530 | 0.32 (0.16–0.65) | **0.002** | ****** |
| **Negative impact on partner (ref: Immensely)** | 527 |  |  |  |
| A lot | 527 | 0.61 (0.25–1.47) | 0.279 |  |
| Moderately | 527 | 0.54 (0.23–1.21) | 0.140 |  |
| Slightly | 527 | 0.46 (0.20–0.99) | 0.053 |  |
| Not at all | 527 | 0.39 (0.18–0.83) | **0.017** | ***** |
| **Mood influence (ref: Always)** | 529 |  |  |  |
| Very often | 529 | 1.04 (0.64–1.70) | 0.882 |  |
| Quite often | 529 | 1.39 (0.84–2.30) | 0.201 |  |
| Seldom | 529 | 0.64 (0.36–1.14) | 0.130 |  |
| Never | 529 | 0.69 (0.29–1.61) | 0.391 |  |
| Complementary/alternative medicine (any) | 530 | 2.00 (1.40–2.87) | **<0.001** | ******* |
| Univariate logistic regression for each predictor independently. Odds ratios (OR) and 95% CIs are shown. N reflects observations with complete data for that predictor.  Sixteen participants with missing data for the primary outcome (pelvic pain evolution after IVF) were excluded from regression analyses; N therefore reflects observations with complete data for both the outcome and the predictor, and may vary across variables due to item-level missingness. | | | | |
| Binary predictors (coded 1 vs 0) are presented on a single line. For multi-level categorical variables, the reference level is shown in parentheses; sub-levels are indented below. 'Unknown' education values were excluded. Bold variable names indicate category header rows. | | | | |
| Abbreviations: rASRM, Revised American Society for Reproductive Medicine; NSAIDs, non-steroidal anti-inflammatory drugs; OR, odds ratio; VAS, visual analogue scale. | | | | |
| *p < 0.05; **p < 0.01; ***p < 0.001. Bold p-values and significance markers indicate statistical significance (p < 0.05). | | | | |

Supplementary Table S2. Complete participant characteristics (all variables) stratified by self-reported pelvic pain evolution after IVF (N = 546).

| **Characteristic** | **Total (N = 546)** | **No worsening (N = 263)** | **Worsening (N = 267)** | **p-value** |
| --- | --- | --- | --- | --- |
|  |  | | | |
| **Socio-demographics** |  | | | |
| Age at survey (years) | 37.7 (6.6) | 37.4 (6.7) | 37.7 (6.4) | 0.419 |
| **Age category** |  |  |  | 0.597 |
| ≤26 | 5 (0.9%) | 2 (0.8%) | 3 (1.1%) |  |
| 27–29 | 28 (5.1%) | 18 (6.8%) | 10 (3.7%) |  |
| 30–32 | 72 (13.2%) | 37 (14.1%) | 33 (12.4%) |  |
| 33–34 | 73 (13.4%) | 35 (13.3%) | 36 (13.5%) |  |
| 35–37 | 121 (22.2%) | 56 (21.3%) | 65 (24.3%) |  |
| 38–40 | 105 (19.2%) | 49 (18.6%) | 53 (19.9%) |  |
| 41–43 | 77 (14.1%) | 38 (14.4%) | 33 (12.4%) |  |
| 44–45 | 26 (4.8%) | 9 (3.4%) | 17 (6.4%) |  |
| ≥46 | 39 (7.1%) | 19 (7.2%) | 17 (6.4%) |  |
| **Geographic region** |  |  |  | **0.018** |
| Europe | 426 (78.0%) | 218 (82.9%) | 197 (73.8%) |  |
| North America | 82 (15.0%) | 34 (12.9%) | 45 (16.9%) |  |
| Other | 38 (7.0%) | 11 (4.2%) | 25 (9.4%) |  |
| **Education level** |  |  |  | 0.469 |
| Primary school | 17 (3.1%) | 9 (3.4%) | 8 (3.0%) |  |
| Secondary education | 101 (18.5%) | 45 (17.1%) | 52 (19.5%) |  |
| Tertiary education | 426 (78.0%) | 207 (78.7%) | 207 (77.5%) |  |
| Unknown | 2 (0.4%) | 2 (0.8%) | 0 (0.0%) |  |
| Out-of-pocket IVF costs | 397 (72.7%) | 190 (72.2%) | 197 (73.8%) | 0.693 |
| Current hormonal treatment | 206 (37.7%) | 98 (37.3%) | 102 (38.2%) | 0.858 |
| **Reproductive history** |  | | | |
| Birth before IVF | 27 (4.9%) | 8 (3.0%) | 18 (6.7%) | 0.069 |
| Age at first IVF cycle (years) | 33.8 (4.6) | 33.3 (4.3) | 34.1 (4.9) | 0.100 |
| Time since first IVF cycle (years) | 3.9 (4.3) | 4.1 (4.6) | 3.7 (3.9) | 0.649 |
| Number of IVF cycles | 2.0 (1.0, 3.0) | 2.0 (1.0, 3.0) | 2.0 (1.0, 3.0) | 0.561 |
| **Reproductive outcomes (totals)** |  | | | |
| Total embryos obtained | 4.0 (2.0, 8.0) | 5.0 (2.0, 9.0) | 4.0 (2.0, 8.0) | 0.120 |
| Total embryo transfers | 2.0 (1.0, 4.0) | 2.0 (1.0, 4.0) | 2.0 (1.0, 3.0) | 0.065 |
| Total pregnancies | 1.0 (0.0, 1.0) | 1.0 (0.0, 1.0) | 0.0 (0.0, 1.0) | **<0.001** |
| **Live births following IVF (total)** |  |  |  | **<0.001** |
| 0 | 350 (64.1%) | 140 (53.2%) | 200 (74.9%) |  |
| 1 | 169 (31.0%) | 104 (39.5%) | 59 (22.1%) |  |
| 2 | 26 (4.8%) | 18 (6.8%) | 8 (3.0%) |  |
| ≥3 | 1 (0.2%) | 1 (0.4%) | 0 (0.0%) |  |
| Number of miscarriages | 0.0 (0.0, 0.0) | 0.0 (0.0, 0.5) | 0.0 (0.0, 0.0) | 0.389 |
| Future pregnancy wish | 375 (68.7%) | 169 (64.3%) | 198 (74.2%) | 1.000 |
| **Endometriosis & surgery** |  | | | |
| **rASRM stage (incl. 'No surgery')** |  |  |  | 0.473 |
| No surgery | 159 (29.1%) | 74 (28.1%) | 78 (29.2%) |  |
| rASRM I | 10 (1.8%) | 5 (1.9%) | 5 (1.9%) |  |
| rASRM II | 27 (4.9%) | 14 (5.3%) | 12 (4.5%) |  |
| rASRM III | 56 (10.3%) | 26 (9.9%) | 29 (10.9%) |  |
| rASRM IV | 207 (37.9%) | 94 (35.7%) | 109 (40.8%) |  |
| Unknown surgery | 87 (15.9%) | 50 (19.0%) | 34 (12.7%) |  |
| Superficial endometriosis | 124 (22.7%) | 64 (24.3%) | 59 (22.1%) | 0.661 |
| Ovarian endometrioma | 206 (37.7%) | 105 (39.9%) | 96 (36.0%) | 0.410 |
| Deep infiltrating endometriosis | 282 (51.6%) | 127 (48.3%) | 148 (55.4%) | **0.021** |
| Adenomyosis | 172 (31.5%) | 84 (31.9%) | 85 (31.8%) | 1.000 |
| Surgery before IVF | 387 (70.9%) | 189 (71.9%) | 189 (70.8%) | 0.848 |
| Surgery after IVF | 166 (30.4%) | 62 (23.6%) | 100 (37.5%) | **<0.001** |
| **Pain measures (current VAS) & menstruation** |  | | | |
| Current pelvic pain (0–10 VAS) | 4.0 (2.0, 6.0) | 3.0 (1.0, 5.0) | 6.0 (3.5, 7.0) | **<0.001** |
| Current dyspareunia (0–10 VAS) | 4.0 (2.0, 6.0) | 4.0 (1.0, 6.0) | 5.0 (3.0, 7.0) | **<0.001** |
| Current menstrual pain (0–10 VAS) | 7.0 (5.0, 8.0) | 6.0 (4.0, 8.0) | 7.0 (5.0, 9.0) | **<0.001** |
| Currently menstruating | 236 (43.2%) | 97 (36.9%) | 132 (49.4%) | **0.004** |
| **IVF cycle events & experience** |  | | | |
| **Most painful step of IVF** |  |  |  | **0.041** |
| Ovarian stimulation (include ultrasound) | 152 (27.8%) | 68 (25.9%) | 81 (30.3%) |  |
| Trigger injection | 30 (5.5%) | 13 (4.9%) | 16 (6.0%) |  |
| Oocyte retrieval | 186 (34.1%) | 105 (39.9%) | 79 (29.6%) |  |
| Embryo-transfer | 30 (5.5%) | 13 (4.9%) | 17 (6.4%) |  |
| First phase of pregnancy | 18 (3.3%) | 9 (3.4%) | 9 (3.4%) |  |
| I don't know | 48 (8.8%) | 25 (9.5%) | 15 (5.6%) |  |
| 1st menstruation after the oocyte retrieval | 82 (15.0%) | 30 (11.4%) | 50 (18.7%) |  |
| Immediate post-IVF cycle worsening (flare) | 284 (52.0%) | 83 (31.6%) | 196 (73.4%) | **<0.001** |
| Unbearable pain during IVF | 54 (9.9%) | 22 (8.4%) | 31 (11.6%) | 0.247 |
| Any complication during IVF cycle | 92 (16.8%) | 40 (15.2%) | 49 (18.4%) | 0.354 |
| Hemoperitoneum | 13 (2.4%) | 9 (3.4%) | 4 (1.5%) | 0.171 |
| Ileus | 5 (0.9%) | 2 (0.8%) | 3 (1.1%) | 1.000 |
| Other complication | 50 (9.2%) | 20 (7.6%) | 27 (10.1%) | 0.360 |
| Unknown complication | 1 (0.2%) | 1 (0.4%) | 0 (0.0%) | 0.496 |
| Live birth after IVF (index cycle) | 196 (35.9%) | 123 (46.8%) | 67 (25.1%) | **<0.001** |
| **Comorbidities** |  | | | |
| Psychiatric disorder (any) | 235 (43.0%) | 106 (40.3%) | 121 (45.3%) | 0.290 |
| Depression | 150 (27.5%) | 65 (24.7%) | 80 (30.0%) | 0.205 |
| Anxiety disorder | 158 (28.9%) | 65 (24.7%) | 88 (33.0%) | **0.044** |
| Bladder pain syndrome | 32 (5.9%) | 7 (2.7%) | 23 (8.6%) | **0.004** |
| Irritable bowel syndrome | 124 (22.7%) | 48 (18.3%) | 72 (27.0%) | **0.017** |
| Migraine | 137 (25.1%) | 71 (27.0%) | 62 (23.2%) | 0.319 |
| Fibromyalgia | 23 (4.2%) | 7 (2.7%) | 15 (5.6%) | 0.126 |
| Chronic fatigue syndrome | 59 (10.8%) | 25 (9.5%) | 32 (12.0%) | 0.401 |
| Chronic inflammatory bowel disease | 46 (8.4%) | 19 (7.2%) | 25 (9.4%) | 0.432 |
| Chronic back pain | 100 (18.3%) | 46 (17.5%) | 52 (19.5%) | 0.577 |
| **Analgesics (current)** |  | | | |
| Analgesic use (any) | 291 (53.3%) | 106 (40.3%) | 181 (67.8%) | **<0.001** |
| Paracetamol | 135 (24.7%) | 53 (20.2%) | 79 (29.6%) | **0.012** |
| NSAIDs | 224 (41.0%) | 78 (29.7%) | 143 (53.6%) | **<0.001** |
| Strong opioids | 17 (3.1%) | 2 (0.8%) | 14 (5.2%) | **0.004** |
| Weak opioids | 64 (11.7%) | 21 (8.0%) | 42 (15.7%) | **0.007** |
| Other analgesic | 38 (7.0%) | 15 (5.7%) | 22 (8.2%) | 0.307 |
| Complementary/alternative medicine (any) | 202 (37.0%) | 77 (29.3%) | 121 (45.3%) | **<0.001** |
| **Care experience** |  | | | |
| **Satisfaction with services** |  |  |  | **<0.001** |
| Never | 74 (13.6%) | 22 (8.4%) | 49 (18.4%) |  |
| Seldom | 157 (28.8%) | 70 (26.6%) | 85 (31.8%) |  |
| Quite often | 137 (25.1%) | 71 (27.0%) | 66 (24.7%) |  |
| Very often | 108 (19.8%) | 64 (24.3%) | 41 (15.4%) |  |
| Always | 67 (12.3%) | 36 (13.7%) | 26 (9.7%) |  |
| **Negative impact on partner** |  |  |  | 0.116 |
| Immensely | 35 (6.4%) | 11 (4.2%) | 23 (8.6%) |  |
| A lot | 57 (10.4%) | 25 (9.5%) | 32 (12.0%) |  |
| Moderately | 89 (16.3%) | 42 (16.0%) | 47 (17.6%) |  |
| Slightly | 147 (26.9%) | 73 (27.8%) | 70 (26.2%) |  |
| Not at all | 212 (38.8%) | 112 (42.6%) | 92 (34.5%) |  |
| **Mood influence** |  |  |  | 0.061 |
| Always | 109 (20.0%) | 53 (20.2%) | 53 (19.9%) |  |
| Very often | 164 (30.0%) | 79 (30.0%) | 82 (30.7%) |  |
| Quite often | 151 (27.7%) | 62 (23.6%) | 86 (32.2%) |  |
| Seldom | 90 (16.5%) | 53 (20.2%) | 34 (12.7%) |  |
| Never | 29 (5.3%) | 16 (6.1%) | 11 (4.1%) |  |
| Of the 546 participants included in the descriptive analyses, 16 did not answer the primary outcome question (pelvic pain evolution after IVF) and could not be classified into either comparison group. These participants are included in the Total column but excluded from group comparisons and regression analyses.  Data are presented as n (%) for binary and categorical variables, mean (SD) for age, or median (Q1, Q3) for count and VAS variables. Percentages are computed within each column using the total N for that column. | | | | |
| Binary variables are presented as a single line for the affirmative response. For multi-level categorical variables, the header row shows the overall p-value; sub-levels are indented. Bold characteristic names indicate multi-level categorical variable headers. | | | | |
| Abbreviations: rASRM, Revised American Society for Reproductive Medicine; NSAIDs, non-steroidal anti-inflammatory drugs; Q1, first quartile; Q3, third quartile; SD, standard deviation; VAS, visual analogue scale. | | | | |
| Statistical tests: Fisher's exact test for binary variables; chi-squared test for multi-level categorical variables; Wilcoxon rank-sum test for continuous variables. Bold p-values indicate statistical significance (p < 0.05). | | | | |

Supplementary Table S3. Multivariable logistic regression for predictors of dyspareunia worsening after IVF.

| **Predictor** | **OR** | **95% CI Lower** | **95% CI Upper** | **p-value** | **Sig.** |
| --- | --- | --- | --- | --- | --- |
| **Patient characteristics** |  | | | | |
| Education level (ref: Primary school) |  |  |  |  |  |
| Secondary School | 2.37 | 0.64 | 10.49 | 0.217 |  |
| Tertiary Education | 1.92 | 0.56 | 8.10 | 0.329 |  |
| Age at first IVF cycle (per year) | 1.00 | 0.96 | 1.04 | 0.978 |  |
| Time since first IVF cycle (per year) | 1.05 | 0.99 | 1.10 | 0.093 |  |
| Hormonal treatment | 0.93 | 0.61 | 1.42 | 0.745 |  |
| **Comorbidities** |  | | | | |
| Anxiety disorder | 1.45 | 0.90 | 2.34 | 0.123 |  |
| Depression | 1.03 | 0.63 | 1.67 | 0.898 |  |
| Bladder pain syndrome | 2.79 | 1.20 | 6.89 | **0.020** | ***** |
| Irritable bowel syndrome | 0.96 | 0.59 | 1.54 | 0.856 |  |
| **Fertility characteristics** |  | | | | |
| Birth before IVF | 1.88 | 0.77 | 4.68 | 0.167 |  |
| Number of live births | 0.93 | 0.63 | 1.36 | 0.701 |  |
| Number of miscarriages | 0.91 | 0.62 | 1.31 | 0.603 |  |
| Number of IVF cycles | 1.02 | 0.88 | 1.18 | 0.822 |  |
| Complications during IVF | 1.64 | 0.73 | 3.64 | 0.225 |  |
| **Endometriosis characteristics** |  | | | | |
| rASRM stage (ref: Stage I) |  |  |  |  |  |
| Stage II | 0.37 | 0.07 | 1.81 | 0.224 |  |
| Stage III | 0.50 | 0.10 | 2.35 | 0.380 |  |
| Stage IV | 0.39 | 0.08 | 1.73 | 0.220 |  |
| Unknown | 0.27 | 0.06 | 1.15 | 0.079 |  |
| Deep infiltrating endometriosis | 1.27 | 0.68 | 2.43 | 0.455 |  |
| No surgery | 0.51 | 0.12 | 2.05 | 0.351 |  |
| **Pain characteristics** |  | | | | |
| Most painful IVF step (ref: Ovarian stimulation) |  |  |  |  |  |
| Trigger injection | 1.02 | 0.38 | 2.56 | 0.968 |  |
| Oocyte retrieval | 1.19 | 0.72 | 1.97 | 0.510 |  |
| Embryo transfer | 1.17 | 0.46 | 2.87 | 0.729 |  |
| Early pregnancy | 3.29 | 1.06 | 10.42 | **0.039** | ***** |
| First menstruation after retrieval | 1.39 | 0.73 | 2.62 | 0.315 |  |
| Don't know | 0.47 | 0.16 | 1.22 | 0.144 |  |
| Unbearable pain during IVF | 0.66 | 0.25 | 1.78 | 0.411 |  |
| Immediate post-cycle pain worsening (flare) | 3.17 | 2.07 | 4.90 | **<0.001** | ******* |
| Results from multivariable logistic regression. Odds ratios (OR) and 95% CIs are shown. Analyses were performed using complete cases (N = 519). | | | | | |
| Reference categories: primary school education, rASRM stage I (endometriosis stage), and ovarian stimulation (most painful IVF step). Age at first IVF cycle was mean-centred prior to inclusion. For binary variables, the reference category is absence of the characteristic (No); for continuous variables, the OR reflects a one-unit increase. | | | | | |
| Abbreviations: rASRM, Revised American Society for Reproductive Medicine; OR, odds ratio. | | | | | |
| *p < 0.05; **p < 0.01; ***p < 0.001. Bold p-values and significance markers indicate statistical significance (p < 0.05). | | | | | |

Supplementary Table S4. Multivariable logistic regression for predictors of dysmenorrhoea worsening after IVF.

| **Predictor** | **OR** | **95% CI Lower** | **95% CI Upper** | **p-value** | **Sig.** |
| --- | --- | --- | --- | --- | --- |
| **Patient characteristics** |  | | | | |
| Education level (ref: Primary school) |  |  |  |  |  |
| Secondary School | 0.22 | 0.03 | 1.58 | 0.133 |  |
| Tertiary Education | 0.33 | 0.05 | 2.06 | 0.233 |  |
| Age at first IVF cycle (per year) | 1.01 | 0.94 | 1.08 | 0.848 |  |
| Time since first IVF cycle (per year) | 1.09 | 0.98 | 1.22 | 0.125 |  |
| Hormonal treatment | 0.68 | 0.30 | 1.48 | 0.327 |  |
| **Comorbidities** |  | | | | |
| Anxiety disorder | 1.09 | 0.50 | 2.41 | 0.827 |  |
| Depression | 0.94 | 0.42 | 2.11 | 0.884 |  |
| Bladder pain syndrome | 2.30 | 0.58 | 11.78 | 0.266 |  |
| Irritable bowel syndrome | 0.96 | 0.41 | 2.25 | 0.925 |  |
| **Fertility characteristics** |  | | | | |
| Birth before IVF | 1.34 | 0.28 | 7.49 | 0.717 |  |
| Number of live births | 0.47 | 0.23 | 0.89 | **0.027** | ***** |
| Number of miscarriages | 0.68 | 0.37 | 1.20 | 0.193 |  |
| Number of IVF cycles | 1.07 | 0.88 | 1.32 | 0.501 |  |
| Complications during IVF | 0.67 | 0.15 | 2.82 | 0.585 |  |
| **Endometriosis characteristics** |  | | | | |
| rASRM stage (ref: Stage I) |  |  |  |  |  |
| Stage II | 1.57 | 0.12 | 17.91 | 0.718 |  |
| Stage III | 1.06 | 0.08 | 12.67 | 0.963 |  |
| Stage IV | 1.18 | 0.10 | 12.11 | 0.888 |  |
| Unknown | 1.19 | 0.10 | 11.35 | 0.881 |  |
| Deep infiltrating endometriosis | 0.98 | 0.38 | 2.54 | 0.971 |  |
| No surgery | 0.87 | 0.08 | 7.70 | 0.902 |  |
| **Pain characteristics** |  | | | | |
| Most painful IVF step (ref: Ovarian stimulation) |  |  |  |  |  |
| Trigger injection | 0.70 | 0.16 | 2.84 | 0.620 |  |
| Oocyte retrieval | 0.66 | 0.29 | 1.49 | 0.322 |  |
| Embryo transfer | 1.14 | 0.34 | 3.91 | 0.838 |  |
| Early pregnancy | 0.14 | 0.01 | 1.59 | 0.148 |  |
| First menstruation after retrieval | 1.74 | 0.67 | 4.64 | 0.260 |  |
| Don't know | 0.71 | 0.18 | 2.58 | 0.606 |  |
| Unbearable pain during IVF | 0.96 | 0.17 | 5.64 | 0.960 |  |
| Immediate post-cycle pain worsening (flare) | 4.03 | 2.08 | 8.05 | **<0.001** | ******* |
| Results from multivariable logistic regression. Odds ratios (OR) and 95% CIs are shown.  Analyses were performed using complete cases (N = 229). The smaller sample size reflects restriction to participants who were currently menstruating and therefore eligible to report dysmenorrhoea, and who had complete data for the outcome and all predictors included in the model. | | | | | |
| Reference categories: primary school education, rASRM stage I (endometriosis stage), and ovarian stimulation (most painful IVF step). Age at first IVF cycle was mean-centred prior to inclusion. For binary variables, the reference category is absence of the characteristic (No); for continuous variables, the OR reflects a one-unit increase. | | | | | |
| Abbreviations: rASRM, Revised American Society for Reproductive Medicine; OR, odds ratio. | | | | | |
| *p < 0.05; **p < 0.01; ***p < 0.001. Bold p-values and significance markers indicate statistical significance (p < 0.05). | | | | | |
