# Supplementary material for: IVF in endometriosis: emerging evidence of exacerbation of pelvic pain and potential predictors
Source: Hum Reprod Open. 2026 Mar 27;2026(2):hoag027. doi: 10.1093/hropen/hoag027 (PMC13091650; doi:10.1093/hropen/hoag027)
Supplement: hoag027_Supplementary_Data [file hoag027_supplementary_data.zip › Supplementary_Figure_S1.pdf]

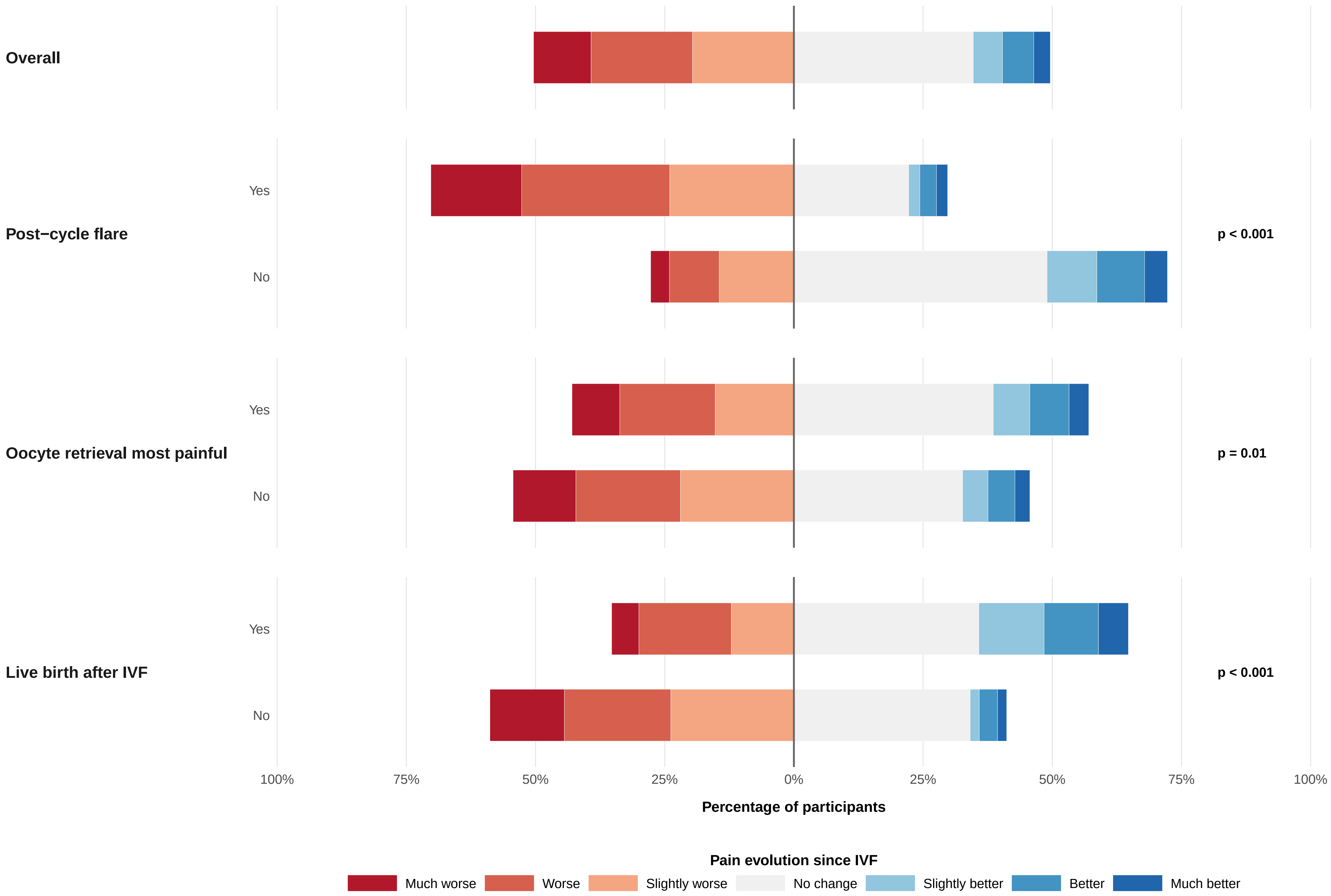

Supplementary Figure S1. Pelvic pain evolution since IVF across clinical subgroups.

Diverging stacked bar chart showing the distribution of self−reported changes in pelvic pain after IVF across clinical subgroups: post−cycle flare, oocyte retrieval perceived as the most painful step, and live birth after IVF. Responses were recorded on a 7−point Likert scale ranging from "much worse" to "much better". Worsening responses (Likert 5−7) are displayed on the left side, while no worsening (Likert 1−4) are displayed on the right side. P−values were obtained using Fisher's exact test comparing worsening vs no worsening.
